# From invisibility to readability: Recovering the ink of Herculaneum

**DOI:** 10.1371/journal.pone.0215775

**Published:** 2019-05-08

**Authors:** Clifford Seth Parker, Stephen Parsons, Jack Bandy, Christy Chapman, Frederik Coppens, William Brent Seales

**Affiliations:** 1 Department of Computer Science, College of Engineering, University of Kentucky, Lexington, Kentucky, United States of America; 2 Bruker Micro-CT, Kontich, Belgium; Politechnika Krakowska im Tadeusza Kosciuszki, POLAND

## Abstract

The noninvasive digital restoration of ancient texts written in carbon black ink and hidden inside artifacts has proven elusive, even with advanced imaging techniques like x-ray-based micro-computed tomography (micro-CT). This paper identifies a crucial mistaken assumption: that micro-CT data fails to capture *any* information representing the presence of carbon ink. Instead, we show new experiments indicating a subtle but detectable signature from carbon ink in micro-CT. We demonstrate a new computational approach that captures, enhances, and makes visible the characteristic signature created by carbon ink in micro-CT. This previously “unseen” evidence of carbon inks, which can now successfully be made visible, is a discovery that can lead directly to the noninvasive digital recovery of the lost texts of Herculaneum.

## Introduction

While written language emerged just 5,000 years ago [1], the vast majority of manuscripts available for study and analysis are only about half this age, with many primary editions having been lost to the turbulence of the ancient era or the deleterious passage of time. Damage and decay, dual realities of the physical world, are constantly at work to rob us of humanity’s written record. Today, our only glimpse of many of our most important texts is through secondary witnesses, painstakingly preserved by medieval scholars, but now teetering on the verge of oblivion.

In the past, no feasible way to overcome this damage existed. Today, however, noninvasive advanced imaging techniques provide a viable solution. In 2015, we used micro-computed tomography (micro-CT) coupled with specialized analysis software—a process we term “virtual unwrapping”—to image and digitally recover a burned Hebrew scroll so fragile that traditional restoration methods were not attempted [2]. For the first time ever, a complete text was digitally retrieved and recreated from inside an object so severely damaged that it would never be physically opened.

This technical breakthrough in the restoration of friable manuscripts forged a pathway for reading the most iconic and inaccessible of irreparably damaged materials: the lost texts of Herculaneum. Buried and carbonized in the eruption of Mount Vesuvius in 79 CE, this cache of more than 1,800 papyrus rolls was found in a luxurious Roman home believed to belong to the family of Julius Caesar’s father-in-law [3]. The collection represents the only large-scale library to have survived from Greco-Roman antiquity and the only classical one to have been found *in situ*. While any successful recovery of humanity’s collective textual record stands to impact the literary and historical canon, the materials from Herculaneum are especially intriguing to scholars [4].

Unfortunately, the Herculaneum papyri represent a perfect storm of challenges for the virtual unwrapping process. They are massively damaged, extremely fragile, and written in a carbon ink known as “lamp black.” While micro-CT overcomes concerns regarding fragility and the threat of additional damage due to physical handling, it also constrains the restoration process, particularly with regards to ink detection. Conventional wisdom claims that “what you see is what you get” in micro-CT. If text doesn’t readily appear when the raw data is analyzed and rendered as a viewable image, then the ink signal must be absent from the data. Such thinking has led to the belief that carbon inks, such as the lamp black of the Herculaneum scrolls, are completely “invisible” to regular micro-CT imaging [5].

In this paper, we refute those claims and conclusively demonstrate the detectability of carbon inks in micro-CT. Previous attempts to render carbon-inked text from micro-CT relied upon a single characteristic of the manuscript to detect the ink: the presence of a relative density difference between the ink and the writing substrate. We show, however, that inked writing substrates demonstrate other characteristic signals that enable the detection of carbon ink in the absence of relative density differences.

Using our knowledge of these characteristics, we construct a machine learning (ML) pipeline, built upon a 3D Convolutional Neural Network (3DCNN), that detects carbon ink in micro-CT data. Our approach does not depend upon the presence of any particular ink characteristic, but rather constructs a general model of the ink’s signal from the micro-CT data. We also construct a modification of this pipeline that generates *photorealistic renderings* of the inked papyrus surface. Finally, we apply both ML pipelines to authentic Herculaneum material, opening the path forward for reading the entirety of the hidden layers and folds of the Herculaneum papyri.

### Related work

Technological progress toward the noninvasive recovery of text from inside damaged manuscripts has occurred rapidly in recent years. The first micro-CT scans of Herculaneum scrolls were performed in 2009, revealing a complex internal structure but an incomplete understanding of the ink signal [6]. Seales et al. proposed the use of phase contrast micro-CT (XPCT), an extension to micro-CT that enhances the contrast between materials with subtle density variations [7]. Subsequent applications of XPCT to Herculaneum scrolls claimed successful ink detection and the identification of individual Greek characters and words [8, 9]. These results, however, have yet to be confirmed, extended, or used to reveal complete passages of text. In fact, no identifiable, readable text has been recovered from inside a closed Herculaneum scroll.

The work on the En-Gedi Scroll [2] represented the first complete, noninvasive recovery of text from inside a badly damaged manuscript. This work formalized the concept of virtual unwrapping, a computational pipeline that converts volumetric datasets into two-dimensional images of recovered texts. Other work in the area of virtual unwrapping has primarily focused on the segmentation and flattening of writing substrates [10–12]. The findings in all of these projects relied on the fact that the revealed ink was significantly denser than the writing substrate and could be seen by filtering the data for bright pixel values.

Most prior work on *carbon ink* recovery has focused on applying various imaging methods so that the ink is directly and clearly visible in the resulting image data. The recent work by Gibson et al. [5] thus far represents the most extensive effort to evaluate the performance of noninvasive imaging methods at capturing different types of ink. Their results discounted the ability of micro-CT to detect carbon ink hidden within closed manuscripts, noting that “XMT [micro-CT] can be used to examine the physical construction of the artifact, and identify heavy metal-based inks, but not carbon-based inks.” They concluded that, “At the moment, there cannot be confidence that deeper carbon-based inks can be detected.”

In recent years, there has been a boom in machine learning research due to the advent of affordable GPU-based computation and the introduction of easy-to-use development tools, such as TensorFlow [13]. It is now easier and faster than ever to train custom machine learning models to handle a variety of previously unexplored problems. Machine learning is widely used for classification tasks that differentiate images according to their features. Deep convolutional neural networks have proven tremendously successful in object recognition using 2D images [14]. ML applications to biomedical images demonstrated remarkable classification performance despite there being only subtle differences among inputs [15, 16].

The development of three-dimensional convolutional neural networks [17] opened an ML pathway for exploring volumetric datasets. Many of these works attempted to identify an object contained within a volume based on its 3D shape [18–20]. Work on biomedical volumes has largely focused on object segmentation—isolating voxels that belong to a particular organ or structure from those in the rest of the volume [21–25]. A recent work by De Fauw et al. shows the promise of 3DCNNs in processing volumetric scan data and producing a classification [26].

Our work differs from those cited above in that we are classifying small regions of volumetric data according to the presence of a localized signal. Bruno et al. performed a similar task in 2D that uses a CNN to label regions of x-ray crystallography images for the likely presence of a protein crystal [27]. In 3D, Ghafoorian et al. used multiple CNNs to detect the location of lacunes within MRI brain scans [28]. They use a multi-scale 3DCNN system as a second pass filter for false positive reduction. These approaches have never before been applied to the problem of detecting inks in micro-CT.

Finally, while neural networks have been used extensively for optical character recognition (OCR) [29–32], our work does not fall into this category. The OCR task is concerned with the identification and recognition of specific, visible letterforms and then the conversion of those letterforms into machine-encoded text, such as a plain text file. In contrast, our work transforms CT scans of manuscripts in which ink cannot be seen into images in which the ink is clearly visible. It does not identify letterforms, but rather a 3D structural pattern in the micro-CT volume that indicates the presence of ink. It is a general approach to ink detection that we believe can be used to enhance any ink in micro-CT, not just the carbon ink we explore in this work. Our method, therefore, is not limited in application to a particular language or character set and is just as useful for revealing inked illustrations as it is for revealing text.

## Rethinking visibility

Until recently, very few people cared what the written word might look like in a micro-CT scan. As a result, the properties associated with the way ink appears in micro-CT are not only complex and obscure, but also understudied. In the absence of a more complete investigation into these properties, a convention formed around the use of a simple ink detection technique that revealed some inks in micro-CT scans. By filtering the micro-CT volume for relative density differences between the ink and the writing substrate—those places where the ink and the substrate have dramatically differing chemistries—the ink could be revealed. This idea is quite reasonable and has been used to great effect. For certain inks, such as the iron gall used in many medieval texts, the ink attenuates the x-rays more than the animal skin or papyrus it covers, and thus it appears to be much “brighter” in micro-CT images. By filtering the micro-CT volume for bright values, these inks become immediately visible during visual analysis of the scan output.

While this direct approach works for inks with heavier components, it has not been effective for carbon inks. Applications of this method to scans of the Herculaneum scrolls, for instance, have not immediately revealed text. This “invisibility” occurs because the chemical composition of the substrate is very similar to the carbon ink coating it. As a result, x-rays pass through both the carbon ink and carbonized papyrus in almost exactly the same way. When the micro-CT images are visualized (via volume rendering or slice viewing), there are no observable intensity differences between the ink and the writing substrate to indicate the presence of text. This effect has resulted in the commonly held belief that carbon ink is undetectable, or even “invisible,” in micro-CT.

The use of the term “invisible” is significant as it reveals a central bias in the attempt to detect carbon inks. The reliance on *direct visual inspection* of the raw data muddles the distinction between what the human eye can see during post-scan analysis and what signals are actually captured by micro-CT. While the ink may remain visually indiscernible, or “invisible,” this does not mean that the imaging method has failed to detect the ink. For the ink to be truly “invisible” to the micro-CT scanner—for any evidence of ink to be completely absent from the resulting scan—the x-rays must pass through the ink without being appreciably attenuated. However, a lack of attenuation would contradict the fact that the ink and the writing substrate have similar chemistries. If the substrate attenuates the x-rays, as is evident in all micro-CT studies of manuscripts, so too must the ink.

**Fig 1:**
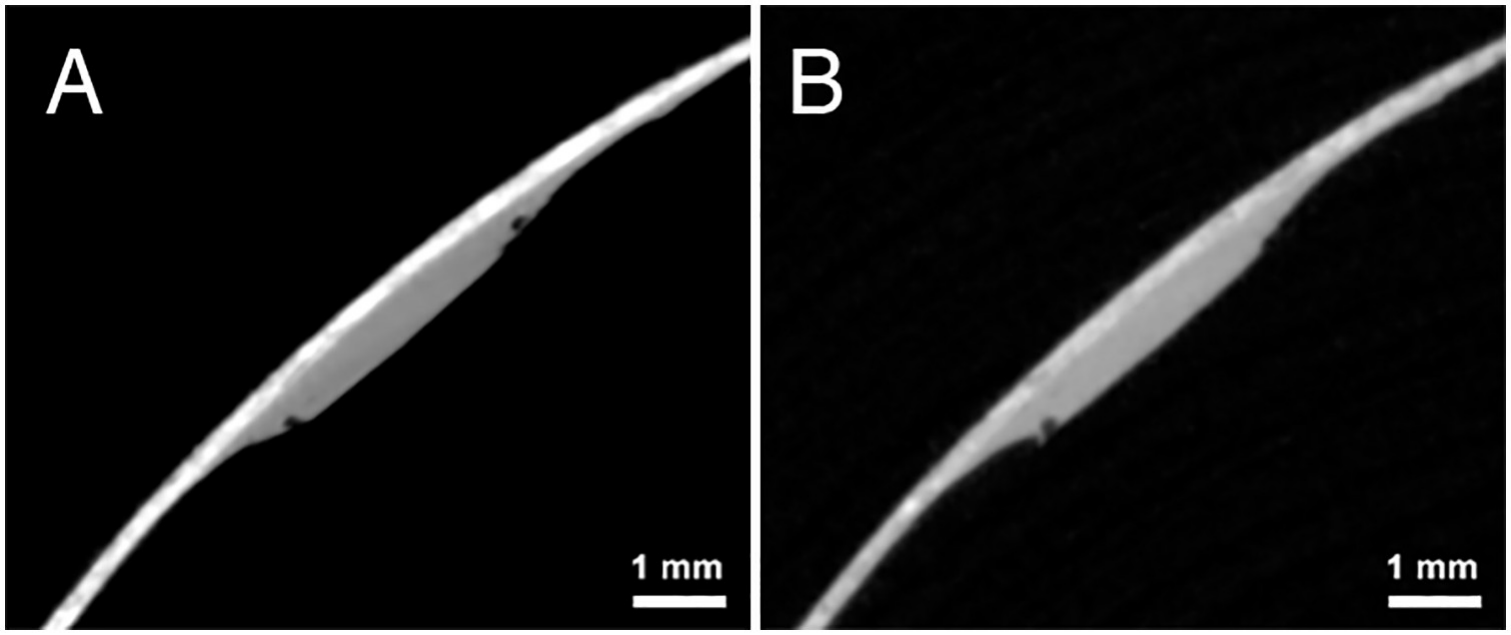
Micro-CT slices of the notecard phantom. A: Low-energy scan (42kV) B: High-energy scan (130kV) The carbon ink can be seen as a pronounced “bump” along the surface in both scans.

In fact, the assertion that carbon ink is “invisible” to the micro-CT scanner is demonstrably false, as shown in Fig 1. For this simple experiment, we acquired a micro-CT scan of a phantom made from a standard office notecard and a carbon ink mixture of lamp black pigment and linseed oil. The ink, generously applied to the notecard, formed small mounds two to three times as thick as the notecard itself. Fig 1 shows the cross-sectional slice images from two different micro-CT scans of the phantom, one low-energy (42kV, 190uA) and one high-energy (130kV, 61uA). These scan settings represent the range of energies that might be used to scan authentic materials *in situ*. In both scans, signal from the ink was captured in the micro-CT scan and is readily visible as a “bump” along the surface of the notecard.

### Contrast

The notecard experiment clearly reveals that micro-CT scanners can capture the presence of carbon ink. Yet, the identification and rendering of that ink during post-scan analysis is a more challenging task. Recognizing the ink signal depends upon the presence of some type of contrast, or discernible difference, between the ink and the substrate. For example, the low-energy scan in the notecard experiment demonstrates *intensity-based contrast*, where the ink and the writing substrate present observably different brightness values.

However, “contrast” need not always be based on intensity. Both scans also demonstrate *morphological contrast*, which is an observable change to the structure or shape of the substrate caused by the application of ink. The cross-sectional bulge in the substrate’s thickness is just this: a shape-based contrast that makes the ink stand out visually.

**Fig 2:**
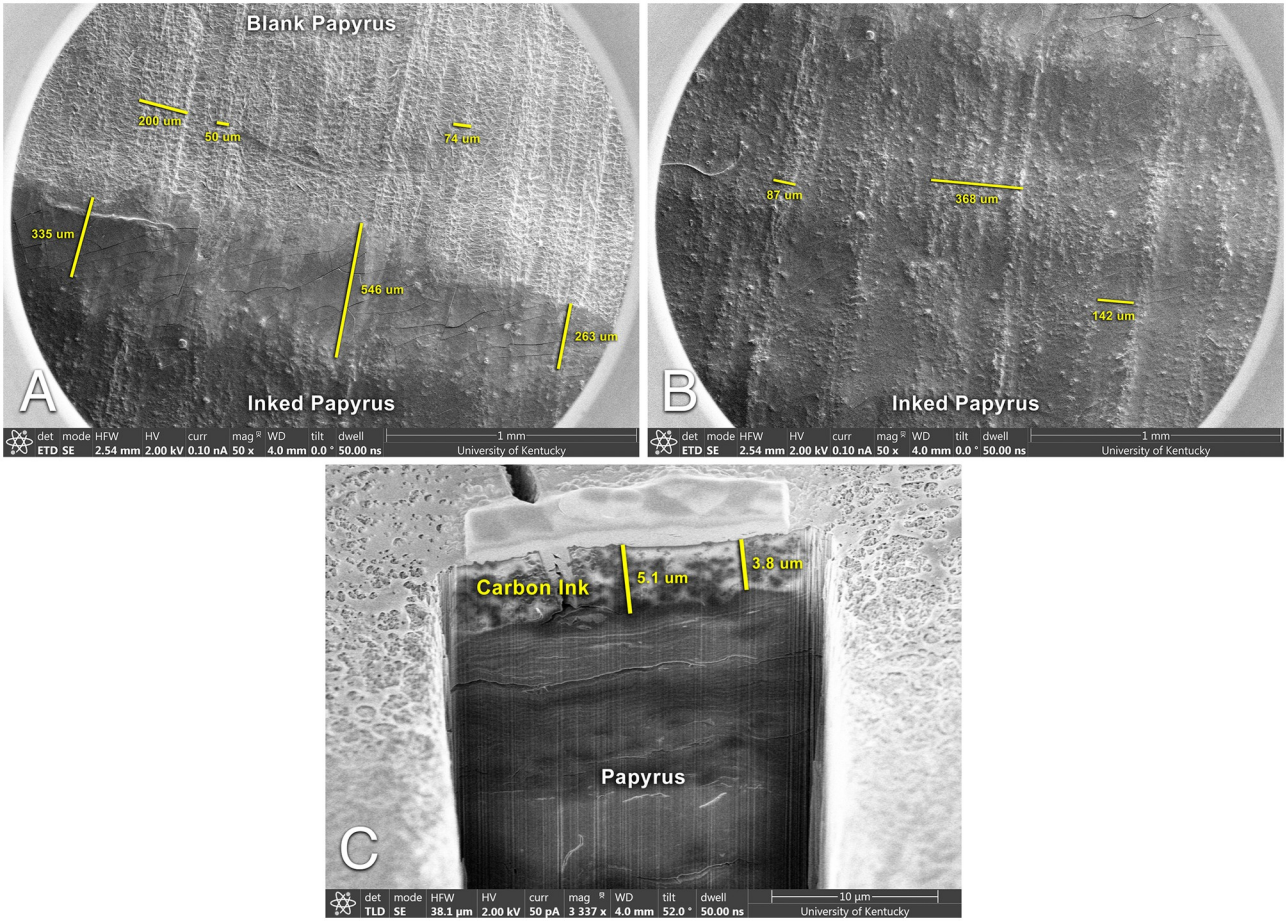
SEM images of a carbon phantom sample. A: Surface SEM image showing both inked and uninked regions. Measurements indicate fiber width, papyrus “cells,” and smooth regions where ink has been applied. B: Surface SEM image showing only an inked region. Measurements show coated fibers and a smooth regions of ink. C: Cross-sectional SEM image of an inked region showing the depth of the ink layer. Depth measurements account for camera angle with respect to the cross-sectional plane.

While the ink in the notecard experiment exhibits an exaggerated morphological profile, such changes also occur when carbon ink is applied at “normal” thicknesses. This effect can be seen in scanning electron microscope (SEM) images of a papyrus phantom coated with a single application of carbon ink (Fig 2). In these images, one can see profound morphological differences between the inked and uninked writing surfaces. The ink coats the writing substrate and modifies the substrate’s structural patterns. These modifications result in a new structural pattern that is unique to the inked writing surface. While some strong surface features remain visible in both regions, the ink-coated surface is visibly smoother than the uninked surface. It also has unique features, such as crack lines, not seen on the uninked surface.

These two types of contrast suggest a holistic approach to ink detection in micro-CT that has thus far gone unexplored. The presence of ink in micro-CT is indicated by a combination of morphological and intensity-based differences spread across the writing surface. These differences may be high contrast (e.g. large intensity differences or a thick ink layer) or they may be low contrast (e.g. little to no intensity difference or a very thin ink layer). By capturing and characterizing the full set of differences between inked and uninked writing surfaces, it is possible to detect and enhance many types of ink, including carbon inks, that have previously gone unnoticed in micro-CT. In this way, we will make the hidden texts of Herculaneum visible once again.

### Scan resolution

With the addition of morphological contrast to the ink detection task, the spatial resolution of the micro-CT scan becomes a critical component to success. While micro-CT captures the presence of the ink in principle, the important morphological features of the ink will be missed by the scanner if the scan resolution is too low. The effective resolution of a micro-CT scan is determined by a number of factors, such as the spot size of the x-ray source and the pixel size of the x-ray detector. For simplicity, we assume that the voxel size of the micro-CT volume is equivalent to the volume’s effective resolution. The voxel size, then, becomes one of the most important scan parameters and must be set in proportion to the size of the ink features.

From the SEM images, we can produce a number of useful measurements that help determine the optimal voxel size. Across the substrate’s surface, the distinguishing features of the ink and papyrus are relatively large. Within a given 300 micron diameter region-of-interest, a dozen or more morphological features exist that may be used to distinguish the inked surface from the uninked surface. In particular, the fibers that comprise papyrus’s characteristic crosshatch pattern form long, continuous “ridges” that vary from 50 to 200 microns in width. Between these ridges, the blank papyrus demonstrates a high-frequency pattern of rounded “cells” that are approximately 50-100 microns in diameter. In the inked papyrus regions, the cells are obscured from view by the ink, while the ridges remain visible but muted.

Cross-sectional SEM images of the inked substrate show that the ink layer is only 3-5 microns thick (Fig 2C). Energy Dispersive x-ray spectroscopy (EDS) analysis of these cross-sections also indicate some absorption of the ink into the papyrus substrate (see S1 Appendix), adding an additional 4-12 microns of depth to the ink signal. Because we cannot assume that ink has been absorbed uniformly across the entire papyrus surface, this results in a total ink layer depth of 3-17 microns.

To ensure that all ink features are represented in the micro-CT data, the smallest measurement related to the ink signal determines the minimum scan resolution required for carbon ink detection. In our measurements, the smallest feature was the depth of the ink layer, which suggests an *ideal* voxel size that is less than or equal to 3 microns. This guarantees at least one voxel sample through the thickness of the ink layer even when there is no ink absorption into the substrate. However, due to the observed variation in ink depth, we limit the voxel size for our experiments between 3 and 17 microns. Voxel sizes larger than 17 microns are not small enough to represent the finely detailed morphology of the ink.

## Methods

We design our two carbon ink detection methods using the observations of the previous section as the foundation: 1) there are morphological and intensity-based differences between inked and uninked writing surfaces and 2) given a small enough voxel size, these differences are captured in micro-CT volumes. Our ink detection methods inspect regions-of-interest (subvolumes) along writing surfaces and use these characteristic differences to determine whether or not carbon ink is present. We do not limit these methods to detecting only one type of contrast, but instead consider all of the differences between the inked and uninked writing surfaces.

The morphological signal that our methods must detect is extremely subtle and non-uniform. The ink layer varies in thickness both above and below the writing surface. Additionally, papyrus is a composite material with a highly irregular surface structure. The differences between any two pieces of papyrus are much more pronounced than the subtle effect of the application of ink. This variability makes it difficult to isolate the ink’s unique contribution to the overall morphological structure of the surface.

**Fig 3:**
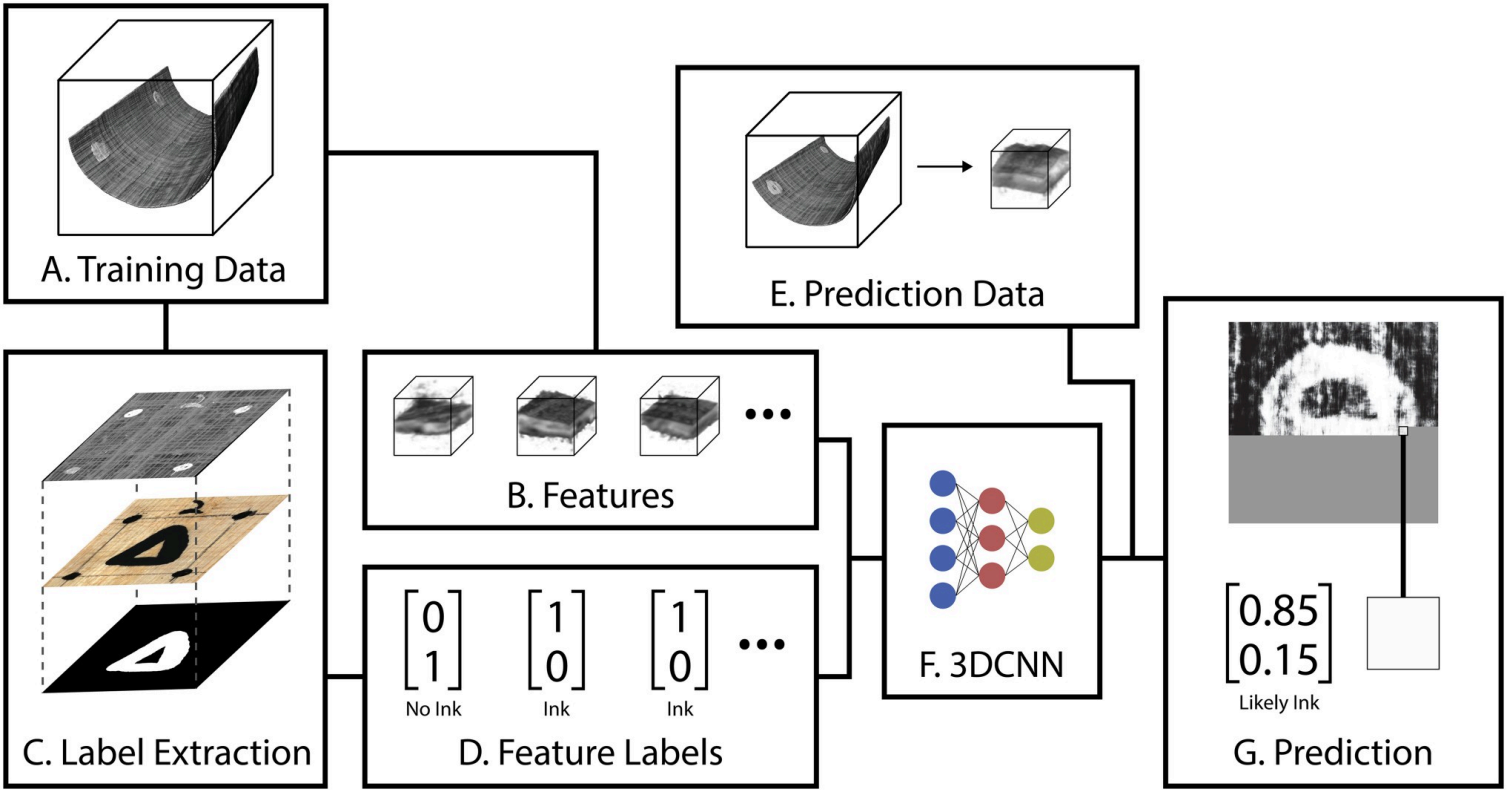
Overview of a system to train a neural network to detect carbon ink in CT scan data.

Our methods address these complexities by relying upon the proven power of supervised machine learning to create a generalized model for ink detection in micro-CT. We train a machine learning model to differentiate subvolumes containing ink from those that do not (Fig 3). Once trained, this model can be applied to subvolumes from previously unencountered micro-CT data to identify and enhance regions that are likely to contain ink.

The model we use for training and classification is a three-dimensional convolutional neural network (3DCNN). Convolutional neural networks are desirable for their ability to build a spatial representation of a signal based upon local features in the data [33]. They have proven remarkably robust for detecting subtle signals in complex and irregular datasets [15, 16].

We have integrated our two methods into our software pipeline for complete virtual unwrapping [2]. This pipeline provides a generic interface for isolating writing surfaces from micro-CT volumes and transforming them into flattened, 2D images of manuscript pages. Integrating our methods with virtual unwrapping avoids the need to process the entire micro-CT volume by focusing computation on only those parts which are near writing surfaces. Within the pipeline, our methods are applied as a texturing module—an algorithm applied after surface isolation and flattening which attempts to detect and render ink. The result of this process is a 2D image which indicates the presence of ink across an entire writing surface (Fig 3G). Our first method produces an “ink prediction image,” which is a black-and-white image where white pixels indicate that ink has been detected. Our second method produces a “photorealistic rendering,” a full color image that simulates what the manuscript might look like under visible light.

### Features and training labels

The input features to our methods are subvolumes from the micro-CT volume that are situated on writing surfaces (Fig 3B). Because of our integration with the virtual unwrapping pipeline, writing surfaces have already been isolated, and extracting subvolumes from these surfaces is straightforward.

Along with the subvolume feature, the 3DCNN also requires a label during training which indicates whether or not a subvolume contains carbon ink (Fig 3D). In many supervised learning tasks, a human operator can generate label information by manually or semi-automatically inspecting the raw input data. This approach will not work for our task, however, because the carbon ink cannot be observed directly in the micro-CT data.

In order to produce this label information, we train our network using scans of reference manuscripts that have been opened, photographed, and inspected for writing. Using the reference photographs that clearly show carbon ink, we generate labels across the entire manuscript surface. For the ink prediction network, these are binary labels of “ink” or “no ink.” We then use the virtual unwrapping pipeline to align these labels with the micro-CT volume, creating a correspondence between the labels and the extracted subvolumes (Fig 3C).

### Photorealistic rendering

Many textual scholars prefer to conduct their analysis with images that appear more like photographs. This propensity is problematic when images are coming from volumetric data that has been generated by x-ray. Many features that are not ink can easily be misinterpreted as such simply by virtue of being brighter or darker than the writing substrate.

To lessen these pitfalls, our “photorealistic rendering network” produces images similar in appearance to full color photographs of opened manuscripts. When applied to an unseen manuscript surface, the result is a color image that estimates how that manuscript might look under visible light. This network differs from our original ink detection network only in the labels used during training. Rather than training the network on binary “ink” or “no ink” labels, we instead train the network on the original RGB values provided by the reference photograph.

### Experimental data

**Fig 4:**
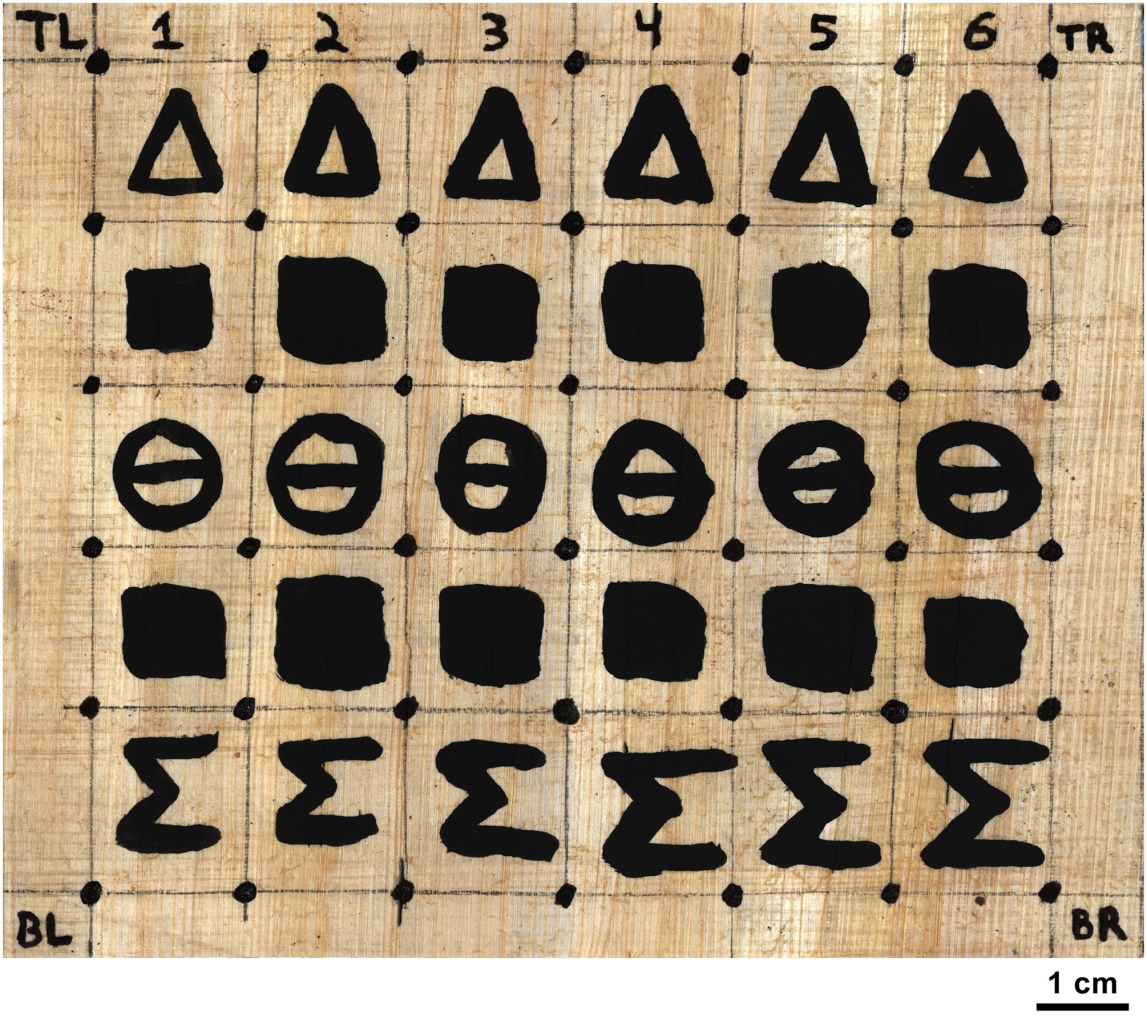
Reference photograph of the opened carbon phantom scroll.

The primary object for this study is a papyrus scroll phantom written in carbon ink, referred to as the “carbon phantom scroll” (Fig 4). This scroll is made from a rolled sheet of modern papyrus that has been divided into a 6x5 grid. Each column of the grid contains the same sequence of five symbols, written using a pure carbon ink mixture. These columns are differentiated by the number of times the ink is applied to the papyrus: Column 1 has a single application of ink, column 2 has two applications, and so on. These graduated applications of ink allow us to test the effectiveness of our method with respect to the voxel size of the micro-CT scan. As the voxel size of the scan gets larger, the resolution of the scan decreases, and it should become more difficult to detect the subtle ink signals in the first few columns of text. Orientation and alignment markers at the corners of the grid, written in iron gall ink, help to locate those regions of the scan that contain carbon ink since the iron gall ink is easily visible in the scan data. Energy-dispersive x-ray spectroscopy (EDS) of the carbon ink surface confirms a lack of iron or lead, two elements which are known to create intensity-based ink contrast in micro-CT. Digital reference photographs and the micro-CT data of the carbon phantom scroll together form the first significant data set for this study. The components of the ink mixture and EDS results for an inked sample are available in S1 Appendix.

**Fig 5:**
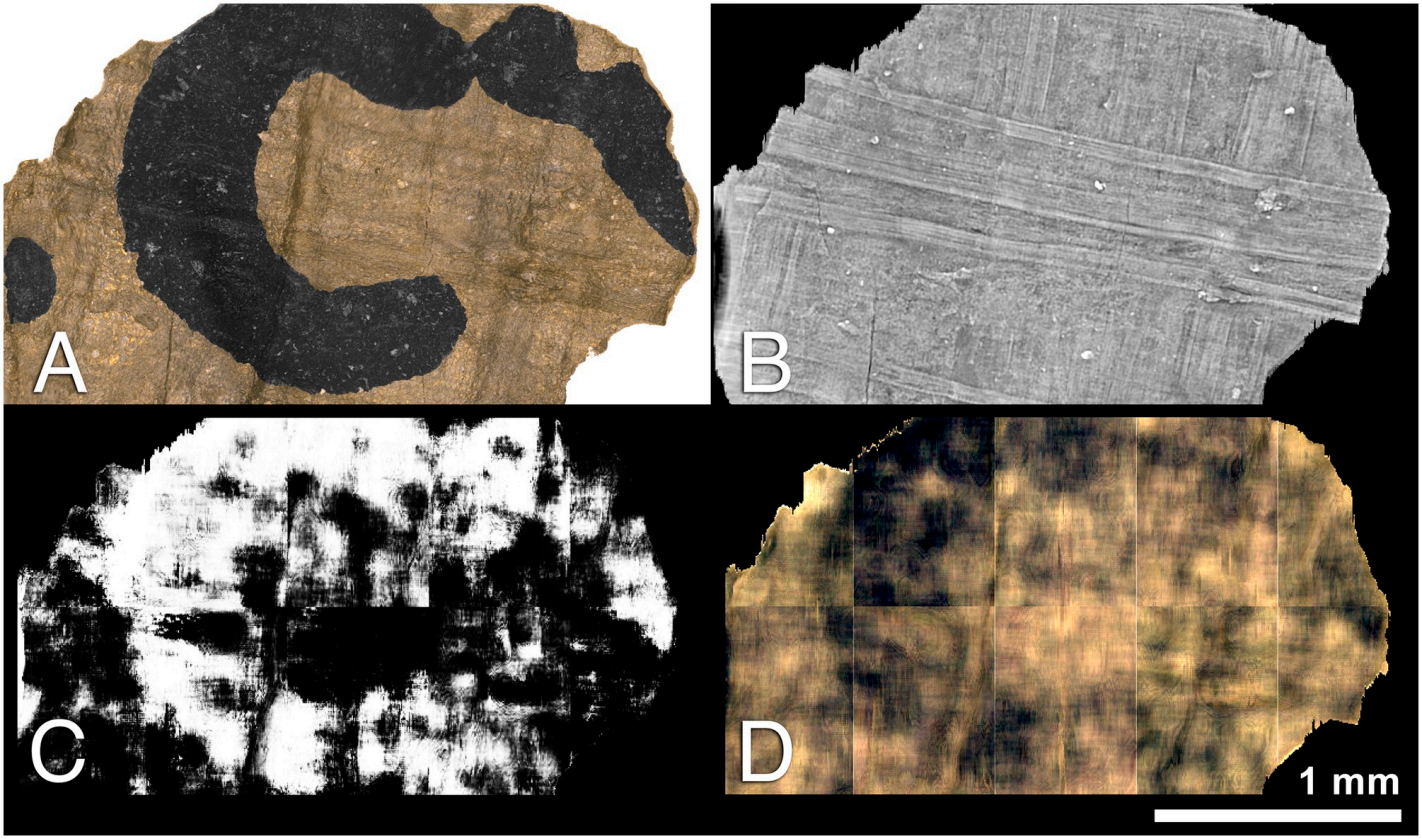
Herculaneum fragment results. A: Reference photograph B: Integral texture image C: Network generated ink prediction image D: Network generated photorealistic rendering.

While the carbon phantom experiment is important for showing the general detectability of carbon inks in micro-CT, it does not answer the question of whether authentic Herculaneum inks can be detected using our methods. In order to confirm that our methods are applicable to Herculaneum material, we also applied them to a tomographic scan of a letter form called the “lunate sigma” (PHerc. Paris 2, Fragment 96; Fig 5A). Our previous study of this and other Herculaneum fragments discovered that “calcium and trace quantities of aluminum, magnesium, strontium and lead are the elements that discriminate between the ink in the Herculaneum fragments and the papyrus alone” [7]. These elements were only present in low concentrations and did not produce intensity-based ink contrast in any of the acquired tomographic data. We cannot rule out the possibility that these elements result in a slight, but currently undetectable, intensity signal where there is ink. However, it is clear that the intensity component alone is not enough to render Herculaneum ink visible in regular micro-CT and that other ink characteristics should be considered as well.

## Results

### Carbon phantom scroll

**Fig 6:**
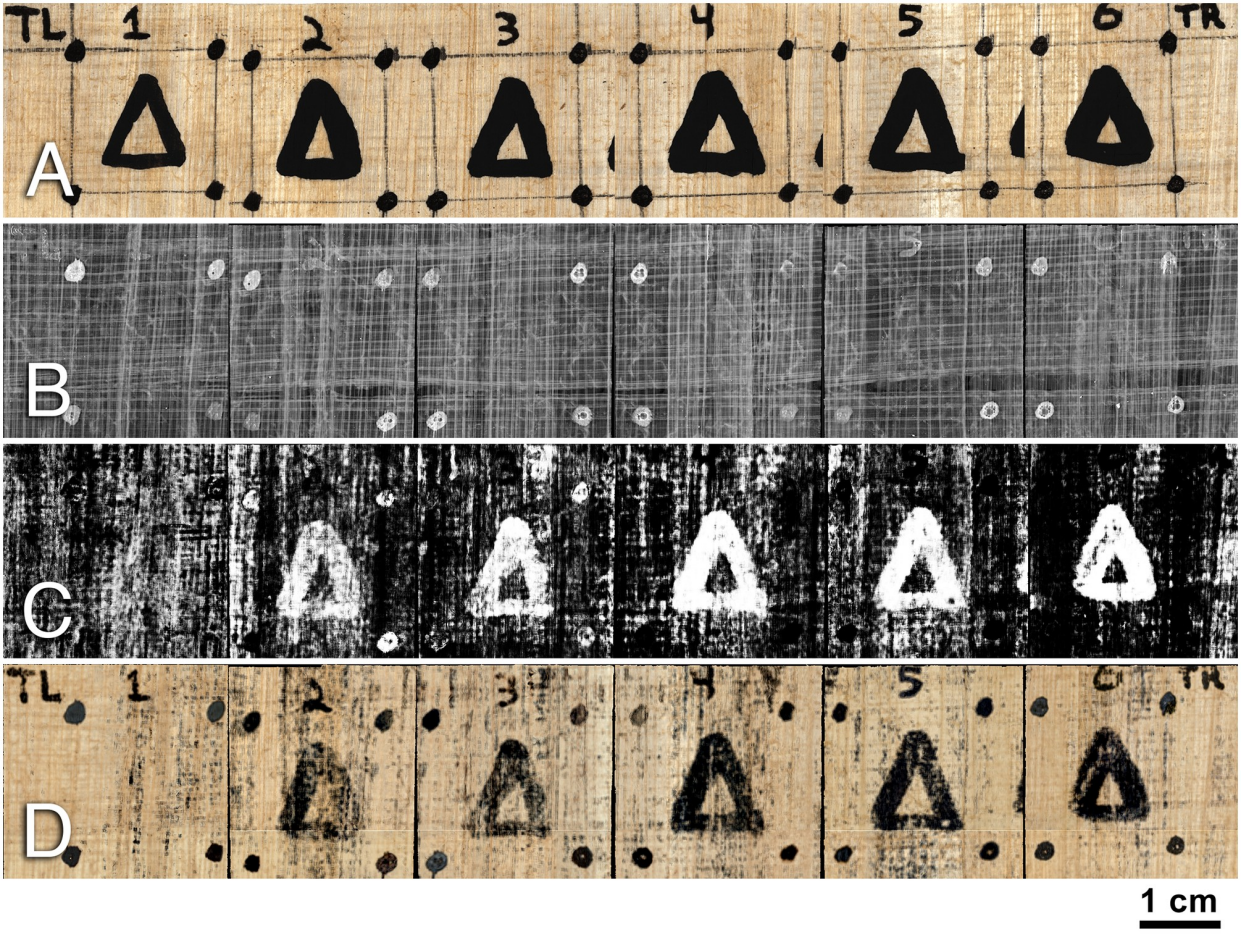
Results for the first row of the carbon phantom scroll. A: Reference photograph B: Integral texture image C: Network-generated carbon ink prediction image D: Network-generated photorealistic rendering.

We applied our two methods to the carbon phantom scroll to produce an ink prediction image and a photorealistic rendering for each of the scroll’s columns. The results for the row of delta symbols are shown in Fig 6. The first image shows a reference photograph of the scroll in which both the carbon ink and iron gall ink are visible. The second image is the result of applying the standard virtual unwrapping pipeline using an integral texturing filter, which represents the former state-of-the-art method for rendering carbon ink from a segmented surface in CT. While the fiber pattern of the papyrus and the iron gall ink are quite clear, the carbon ink delta is only slightly visible in columns 5 and 6. More detail about the rendering process is available in S1 Appendix.

The third image shows the results from applying our ink detection network. One can clearly see that the network detects the carbon ink in the raw CT data where it was previously not visible. It is also obvious that the network performs this function best in the columns where the ink’s morphology is more pronounced.

The final image shows the results of the photorealistic rendering network. In addition to the carbon ink signal, the network demonstrates that it has learned other features of the scroll, such as the iron gall ink and the papyrus fibers, and is capable of expressing these in color.

The carbon ink signal in column 1 is on the border of detectability by both networks. This result is due to the voxel size of the carbon phantom volume. The carbon phantom scroll was scanned at a 12 micron voxel size. While this voxel size falls within our expected bounds for the minimum required voxel size, it approaches the upper limits of the range. The morphological component of the column 1 ink as expressed in micro-CT is simply too small to be adequately captured by a 12 micron voxel size. In contrast, the ink is readily apparent in column 2, which suggests that the network will be able to detect the column 1 ink with only a small increase to scan resolution. The false positives in columns 2 and 3—the places on the writing surface that the network incorrectly identifies as containing ink—indicate that our methods are making use of the morphology contained in the subvolume in order to detect the ink. Many of these false positives align with papyrus fibers, suggesting that the combined thickness of the substrate and the ink plays a role in the network’s determination of ink. This result is supported by the strong response of our network to columns 5 and 6, where the ink’s morphology is the most pronounced. It is easier for the networks to detect the ink because it has a clear morphological difference from the papyrus.

The photorealistic rendering network has similar performance to the standard ink detection network when detecting carbon ink. However, the subjective clarity of the text provided by the photorealistic network appears to be much better. Areas of the surface where both networks produce high false positive rates are visually less noisy in the photorealistic render image than in the ink prediction image. This clarity is due to the additional expressivity provided by having three output channels (R, G, and B) for each pixel in the photorealistic render. This additional information allows many of the false positives generated by the ink detection network to be correctly rendered as fibers by the photorealistic rendering network. Because of the networks’ shared architecture, this suggests that the ink detection network can be improved by introducing labels for surface features other than ink.

**Fig 7:**
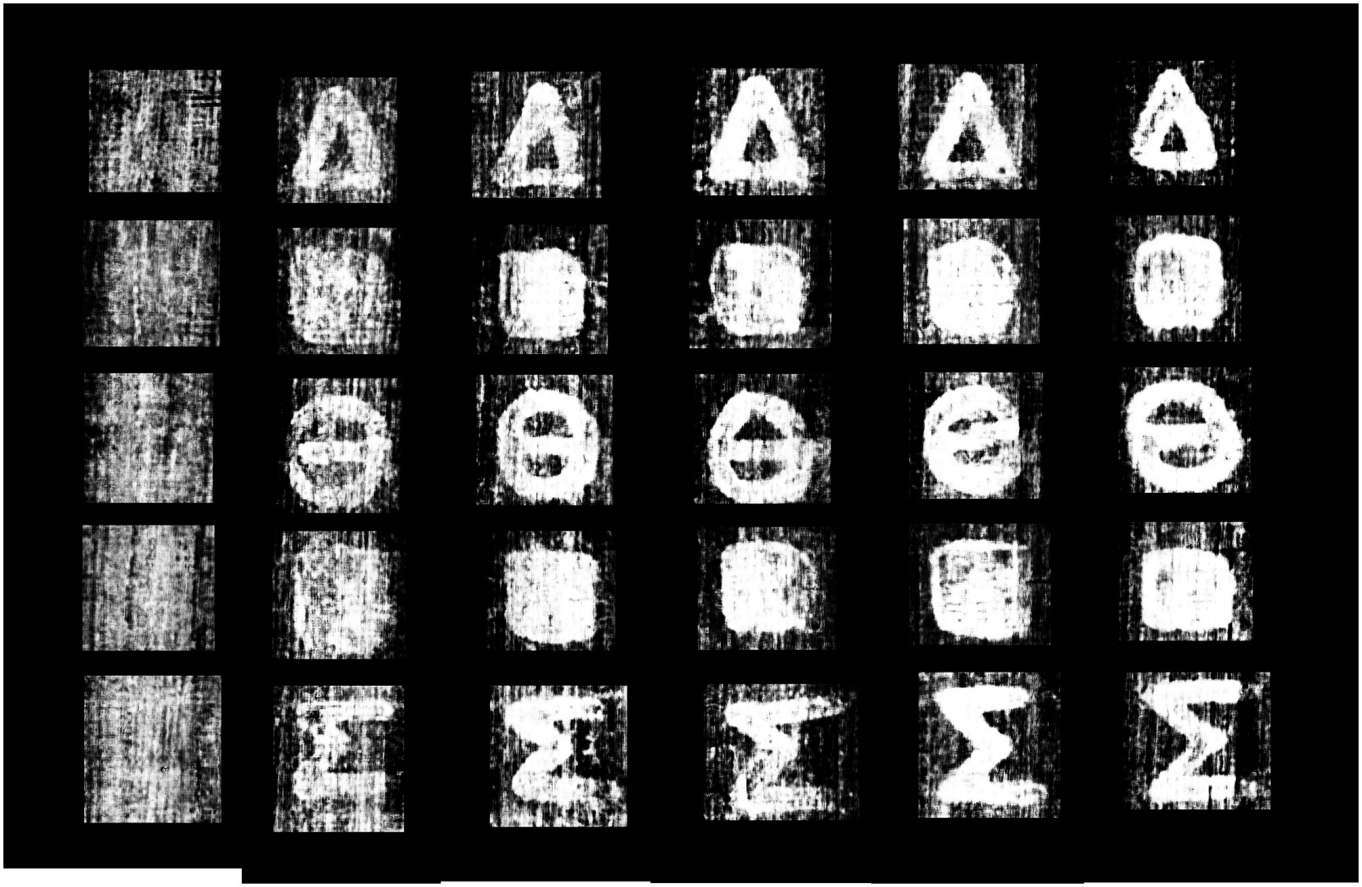
Ink prediction image for the carbon phantom scroll, generated by the ink detection network. This image is comprised of six training runs where one column at a time was isolated as the prediction and evaluation column.

**Fig 8:**
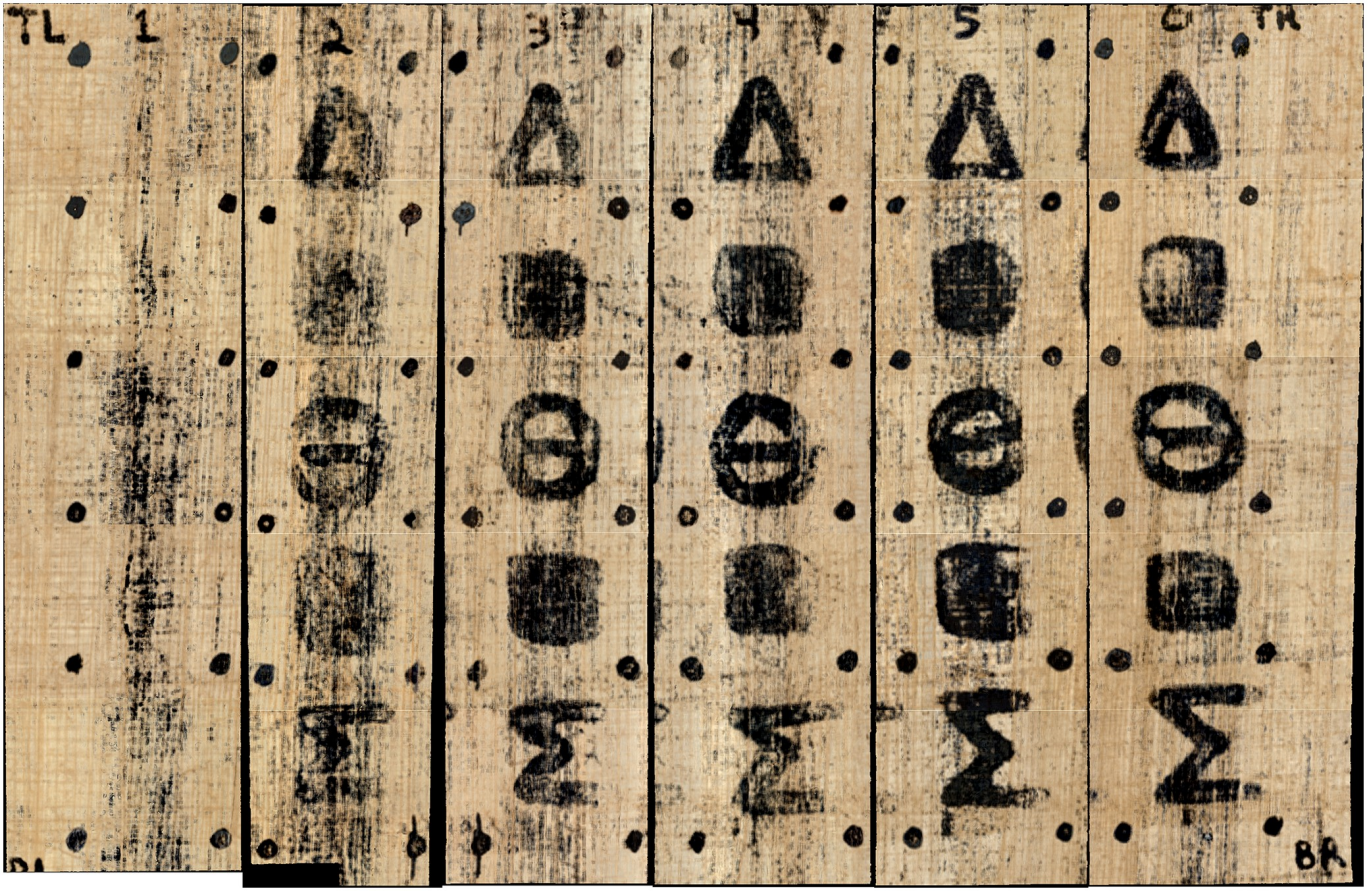
Photorealistic rendering of the carbon phantom scroll. This image is comprised of six training runs where one column at a time was isolated as the prediction and evaluation column.

Full image results for both methods, depicting all columns and rows of the carbon phantom scroll, are shown in Figs 7 and 8.

### Lunate sigma fragment

Fig 5 shows the results generated by applying both networks to the lunate sigma fragment. The first image (Fig 5A) is the reference photograph of the fragment. This image was captured using a confocal microscope; the color was enhanced to highlight the carbon ink. The second image (Fig 5B) is a texture image generated by the standard virtual unwrapping pipeline using an integral filter (described in S1 Appendix). The third image shows the results of applying our ink detection network, and the final one shows the results of applying our photorealistic rendering network.

The small surface area of the fragment required a variant of *k*-fold cross validation in order to train and make predictions on the lunate sigma volume. We separated the dataset into ten non-overlapping regions and produced a network prediction image for each of these regions. The results were then combined into the single prediction images shown in Fig 5C and 5D. More information about this process is available in S1 Appendix.

The results for the lunate sigma fragment clearly show that our methods are capable of detecting carbon ink on authentic Herculaneum fragments. Despite the small number of training examples, the letter form is discernible across the entire composite prediction image. In the lower region of the fragment, in particular, one can see the curved shape delineating the boundary edge of the letter form. The curved shape resulting from the detected ink signal is an important feature since the presence of the curve is unlikely to arise from papyrus fibers, which are linear and orthogonal to each other. No other observable features of the fragment’s surface are curved in this way.

## Discussion

Our work lays an important foundation for the future of digital restoration of damaged texts. We have demonstrated that regular micro-CT can be used to detect carbon inks and that these inks can be revealed for scholarship via a computational approach. This result overturns a prevalent mischaracterization of the power of micro-CT and opens up a pathway for the recovery of texts previously thought inaccessible. Moreover, we have shown that the same computational method used to detect the ink can be used to enhance the visualization of the material, making scholarship easier than ever before. The ability to enhance data across modalities is powerful and not limited to micro-CT volumes and full color photography. In particular, we anticipate that the integration of spectral image data into this pipeline will render this tool even more powerful for scholarship.

Of particular importance is the ability of our network to detect authentic Herculaneum ink. This opens an exciting new pathway for reading the entirety of the Herculaneum scroll collection. We believe that a carbon ink detection network, properly trained on a large reference library of micro-CT scans, can be applied to micro-CT scans of unopened Herculaneum scrolls to reveal previously hidden text.

However, the success of this approach is predicated upon access to authentic materials. The power of our network to detect carbon ink is enabled primarily by the data used to train it. We must build a reference library of micro-CT scans of authentic carbon ink texts, and no better collection exists than that of the fragments from opened Herculaneum scrolls. Scanning and photographing these fragments will provide the necessary training data for creating a robust and powerful ink detection network that will enable the reading of intact Herculaneum scrolls.

A critical component in the design of the reference library is the voxel size at which scans should be acquired. Through these experiments, we have determined that the minimum required voxel size to detect carbon ink in micro-CT is between 3 and 12 microns, likely nearer to the smaller of those values. Our ability to establish this range more precisely is hindered only by the lack of scans of authentic material with this level of detail.

The ability to acquire scans at these voxel sizes is a logistical problem rather than a technical one. Many of the smaller Herculaneum fragments, both those with exposed text and those still wrapped, can be scanned at or near 5 micron voxel sizes using existing laboratory scanners. For larger objects, including the fully intact Herculaneum scrolls, scans can be performed at a synchrotron facility or by constructing a custom micro-CT scanner. Synchrotrons offer extremely high spatial resolutions at much faster per-scan acquisition times when compared to conventional laboratory systems. However, the access and cost restraints of a synchrotron make this option unfeasible for acquiring the large number of scans required to build a complete reference library.

Alternatively, constructing a custom micro-CT system capable of scanning the larger Herculaneum pieces is feasible using existing technology. While many factors go into designing such a system (see “Designing a Custom Micro-CT Scanner” in S1 Appendix), the key challenge in reaching a high-enough spatial resolution is having enough pixels horizontally in the x-ray projection images. Many systems achieve smaller voxel sizes by zooming in on the object and off-setting, or tiling, the x-ray detector to produce a wider image. The Bruker SkyScan 1272, a state-of-the-art laboratory system with a 16 megapixel CCD camera, allows triple off-setting of the sensor. This system could achieve a 6.92 micron image pixel for a Herculaneum scroll with a 10cm diameter. In principle, this approach can be extended by tiling the detector four times to reach a 5 micron pixel size.

While our ink detection methods represent a major step forward in the noninvasive analysis of damaged manuscripts, there are areas for continued development, particularly with regards to the scale of the experiments. Manuscripts vary in their construction and chemistry, and we do not yet know how these differences will affect the appearance of surface ink features in micro-CT. Similarly, micro-CT volumes vary significantly in their scan parameters and in the dynamic range of their voxels. We have not yet explored how sensitive our network is to these parameters, but we anticipate the need to introduce some type of normalization step within our network to account for these differences. We believe that all of these questions have clear answers and that the full potential for our network will be achieved as additional authentic data is acquired.

## Conclusion

While the text within damaged and fragile documents can be stubbornly resistant to any and all efforts to reveal its contents, our work has taken another successful step towards the complete recovery of the world’s most precious manuscripts. Our results overturn the belief that micro-CT offers no solution for carbon ink manuscripts, thereby opening up a new category of texts for noninvasive, digital restoration. Within this category are the Herculaneum scrolls. With our machine learning pipeline’s proven ability to elicit the carbon ink signal and render it in a photorealistic way, the scholarly community may indeed be one step closer to witnessing “a bursting forth of genius from the dust” [34] of Herculaneum.

## Supporting information

S1 Appendix Expanded materials and methods. Detailed descriptions of materials, methods, and computational processes. Includes report on alternative ink detection filters developed prior to the work presented here.
(PDF)
