# Supplementary material for: From invisibility to readability: Recovering the ink of Herculaneum
Source: PLoS One. 2019 May 8;14(5):e0215775. doi: 10.1371/journal.pone.0215775 (PMC6505748; doi:10.1371/journal.pone.0215775)
Supplement: S1 Appendix — Detailed descriptions of materials, methods, and computational processes. Includes report on alternative ink detection filters developed prior to the work presented here. (PDF) [file pone.0215775.s001.pdf]

S1 Appendix.

Materials and methods

Carbon ink recipes The inks used in our phantom experiments were mixed by hand in our lab. They did not follow any particular authentic recipe, but rather were mixed to represent an idealized carbon ink, one that was almost entirely composed of carbon pigment. In our experiments, we were primarily interested in determining whether ink could be detected in micro-CT using its morphological characteristics. For this reason, we wanted to avoid ink mixtures that would form intensity contrasts with the papyrus. We found that a pure carbon ink mixture met this criteria.

The ink used on the notecard phantom scroll was a mixture of lamp black pigment and linseed oil. The ink used on the carbon phantom scroll was a mixture of powderized carbon, gum arabic, and distilled water.

Experimental data acquisition The carbon phantom scroll was scanned using a Bruker Skyscan 1276 with exposure settings of 45kV, 200uA, and 611ms. The volume was reconstructed using NRecon to a 16-bit TIFF stack with a 12 micron voxel size. Reference photographs were taken using a MegaVision E6 multispectral camera system. The full color photograph was created by merging the red (638nm), green (535nm), and blue (465nm) bands from the spectral image set.

The lunate sigma fragment was scanned at the Diamond Light Source I12 beamline in Oxfordshire, UK, at approximately a 1.3 micron voxel size with an incident energy of 53 keV.

SEM/EDS analysis SEM/EDS analysis was performed on an inked papyrus sample prepared using the same materials and methods as the carbon phantom scroll. A ThermoFisher Scientific Helios Nanolab 660 Dual Beam was used for imaging and cross-sectioning of the sample. It is equipped with an Oxford Instruments X-MaxN EDS detector which was used to perform elemental analysis (EDS).

Surface imaging shows a clear morphological delineation between the inked and uninked regions of the papyrus surface (Figs 2A, 2B). The uninked surface is characterized by a continuous network of high-frequency, cell-like structures. These cells are absent from view in the inked areas, having been covered by the thin film of ink. Additionally, EDS analysis of the surface shows higher counts of carbon in the areas where ink has been applied (Fig S1.1). The inked area does not demonstrate contamination from iron or lead, two heavier elements which, if present in sufficient quantities, could introduce an intensity-based contrast between the ink and the papyrus.

Cross-sectional imaging into the depth of the papyrus shows an ink layer that is approximately 3 to 4 microns thick (Fig 2C). EDS analysis of the cross-sectional images also shows high counts of carbon that penetrate for some depth

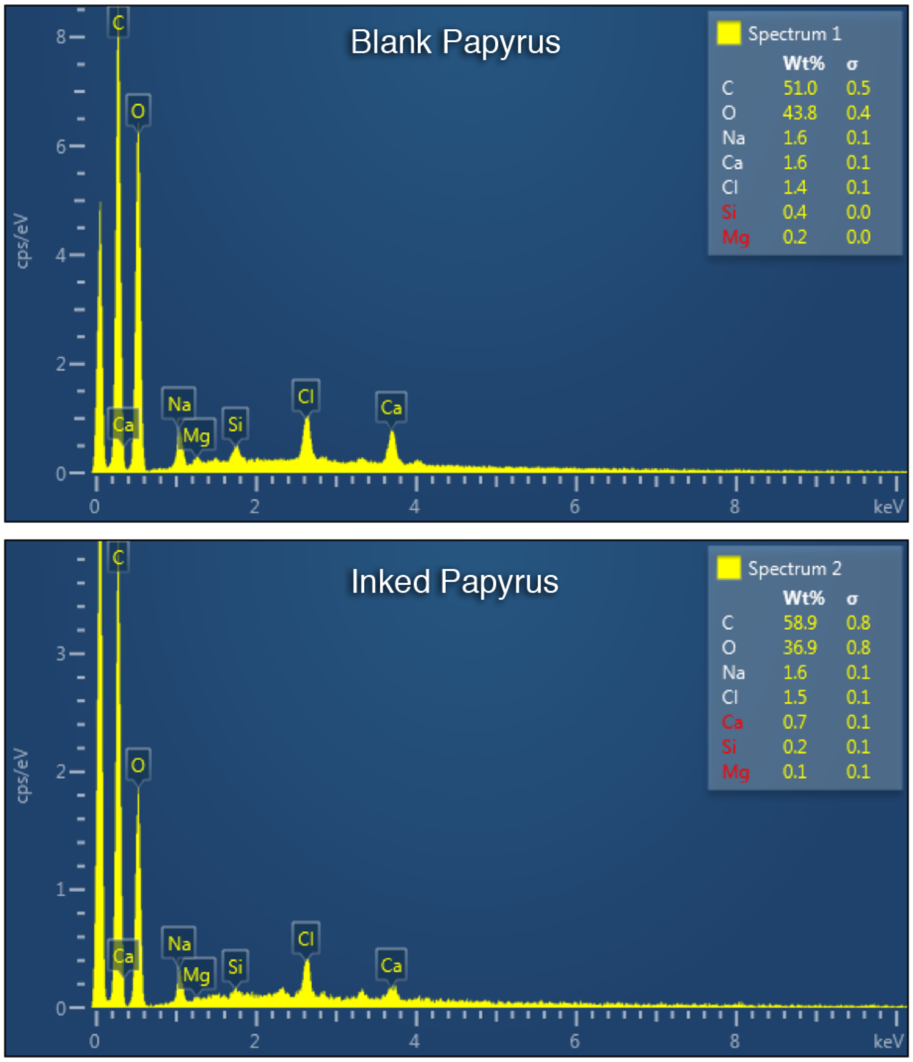

Fig S1.1. EDS analysis of the surface of the carbon phantom. The inked papyrus demonstrates higher counts of carbon than the blank papyrus.

(4-12 microns) into the papyrus layer (Fig S1.2). Given the high counts of carbon observed on the inked surface, this suggests that the ink has been absorbed into the papyrus layer.

Virtual unwrapping preprocessing We make use of our in-house virtual unwrapping software, Volume Cartographer, in order to preprocess all of our datasets into a canonical input form. First, we segment the writing surfaces of our datasets, creating an open-manifold triangular mesh that intersects the

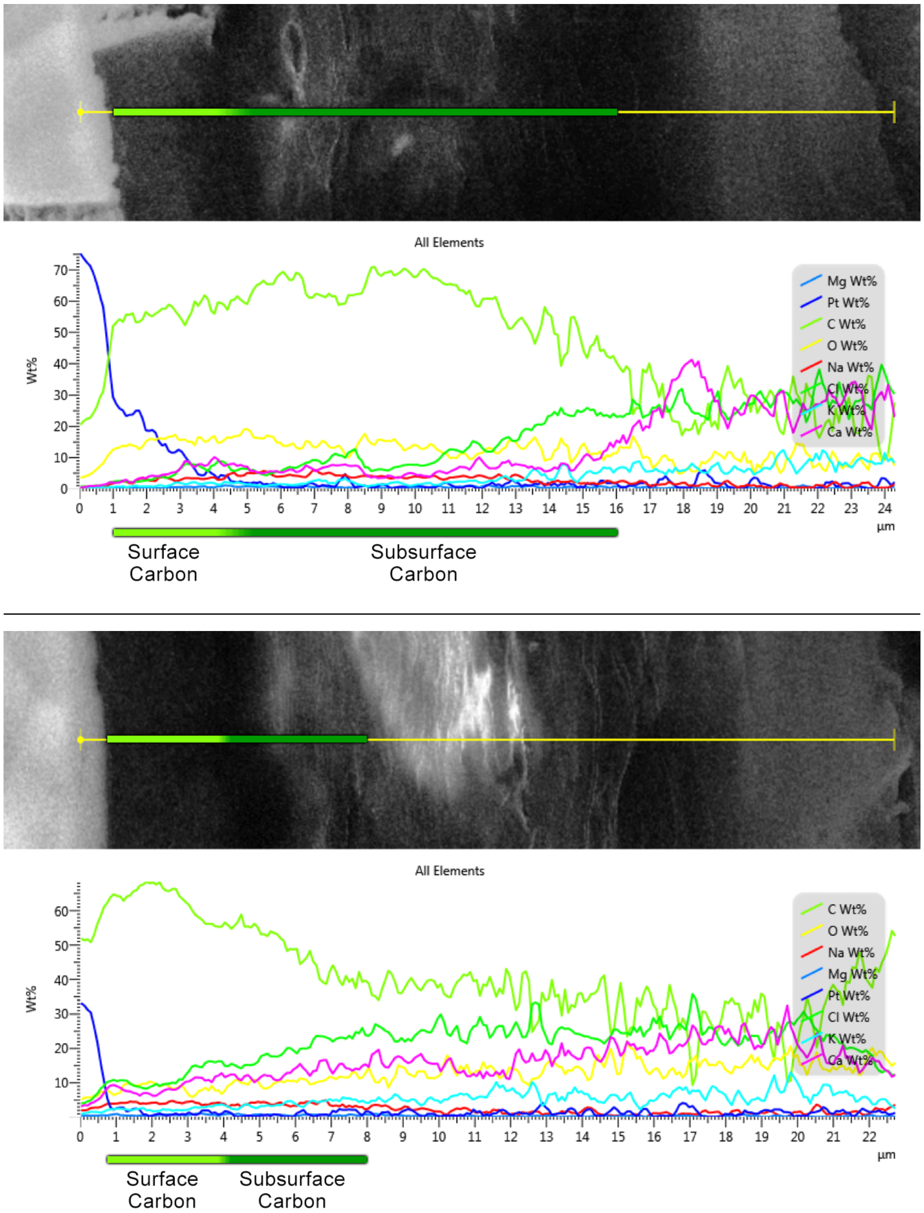

Fig S1.2. EDS analysis of two cross-sections of the carbon phantom. In both, high carbon counts indicate a surface ink layer approx. 4 microns in depth and a subsurface layer of absorbed ink ranging in depth from 4-12 microns.

approximate center of the manuscript's page or layer. In the case of multi-layer objects, multiple surfaces are developed such that each layer is processed independently. These surfaces are uniformly resampled using approximated centroidal voronoi diagrams [1]. The mesh is then flattened using an implementation of ABF++ [2].

We convert the flattened mesh into a texture image by sampling the mesh's surface at unit intervals. This creates a blank texture image. The process for filling the texture image is straightforward: we iterate over the pixels of the texture image, convert the current 2D position into a 3D position using the flattening transformation, filter the volume around that 3D position for ink signal, and place the result into the current pixel. By varying the filter function, we can generate any number of aligned texture images. The two methods presented in this paper are each filter functions that can be deployed at this stage.

Computing the 3D positions for each 2D pixel can be an expensive operation, but the results can be reused by future texturing processes. We store these values in a data structure that we call a "per-pixel map." The per-pixel map is an image with the same dimensions as the texture image. Each pixel stores its own 3D position and surface normal that were computed from the original mesh.

Our existing virtual unwrapping pipeline contains a number of filter functions that we use to explore and examine flattened manuscript layers. Two filters in particular, the maximum intensity filter and the integral filter, were used heavily during this study to create reference images and to aid with the process of label generation. Both perform simple filtering of a given subvolume. The maximum intensity filter returns the maximum-valued pixel within the subvolume. The integral filter returns the sum of all pixel values within the subvolume. Once all pixel values have been calculated, the floating-point integral image is linearly scaled to a 16-bit dynamic range.

Network input features The input features to our neural networks are subvolumes which are extracted from the micro-CT volume and which are situated on writing surfaces (Fig 3B). Because we have preprocessed the data using the virtual unwrapping pipeline, collecting these subvolumes is straightforward. We iterate over the per-pixel map generated by virtual unwrapping to collect a list of all possible surface points. To better generalize the learned model across the set of training data, this list is shuffled prior to network training. Next, we map these points back into the 3D volume, which gives us the center positions of our surface subvolumes.

For this study, we want the subvolumes to have a consistent orientation with respect to the writing surface. Because the subvolume may contain voxels from both the front and the back sides of the writing substrate, consistent orientation helps the network focus on those parts of the substrate where we expect ink signal. Fortunately, we have an estimate of each subvolume's orientation in the form of a surface normal generated by virtual unwrapping. We align the

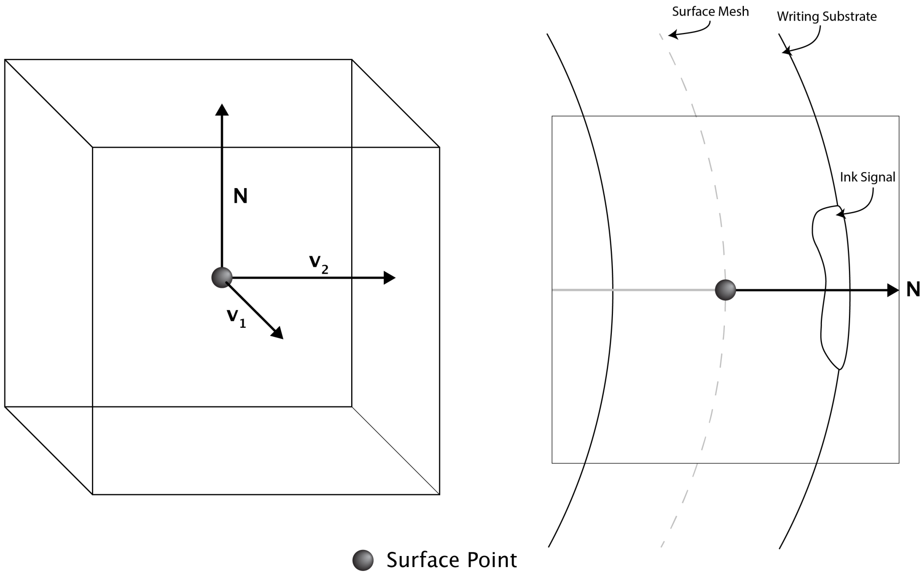

Fig S1.3. Construction parameters for a subvolume feature. (Left) All subvolumes are provided to the network in the pictured orientation. (Right) When extracting intensities from the micro-CT volume, the primary axis of the subvolume, N, is directed towards the expected location of ink.

subvolume's primary axis with this surface normal and generate two additional, mutually orthogonal vectors for the minor axes (Fig S1.3). The side lengths of the subvolume are a runtime parameter when training the network and should be set according to the voxel size of the volume.

To fill the voxels of our subvolume, we estimate subvoxel intensities from the original volume using a nearest neighbor interpolation. We chose this method as it formed the best compromise between input quality and computational cost. An alternative approach would be to use trilinear interpolation, which in our experiments showed a marginal network performance improvement at the cost of a 4x increase in training duration. Another option would be to use an axis-aligned extraction, where the subvolume's primary axis is snapped to the nearest CT volume axis. The subvolume intensities can then be extracted through direct indexing rather than per-point interpolation. This method provides marginally faster training durations than nearest neighbor sampling, but shows reduced network performance when the writing substrate approaches a 45° angle to the CT volume's axes.

During training, we randomly augment the subvolumes in the training data using a variety of linear transformations. As noted by Krizhevsky et al., data augmentation is "the easiest and most common method to reduce overfitting" [3]. Prior to interpolation, we randomly translate the center of the subvolume along the surface normal up to +/-4 voxels. This helps make the network resilient to the irregular thickness of the writing substrate caused by the over-

lapping papyrus fibers. After interpolation, we also randomly flip and rotate the subvolumes around their primary axis. This preserves the consistency of our subvolume orientations while expanding the complexity of the training data set. The subvolumes are then batched before being input into the network.

Training labels We train our networks on scans of manuscripts which have been opened, photographed, and inspected for writing. Because a human is not capable of identifying the carbon ink signal in micro-CT volumes, we developed a system for creating labeling information from photographs of the opened manuscript.

First, we render a texture image of the scroll by running the virtual unwrapping pipeline on the segmented surface. This produces a texture image which does not show the carbon ink but does show many other features that are also visible in the photographs, such as fiber strands and the iron gall orientation markers. Using these features, we align the photographs of the object to the generated texture image using the Insight Segmentation and Registration Toolkit [4]. This aligned photograph is then manually binarized in Photoshop in order to generate per-pixel labels for the carbon ink signal. For the photorealistic rendering network, we skip the binarization step altogether and instead provide the network with the RGB color values from the photograph as the input labels.

Through this process, we create a 1:1 correspondence between the carbon ink label image and the flattened surface. Any subvolume selected during network training can now be correlated with the label image in order to determine whether or not it contains ink.

Network layout Our 3DCNN is implemented in Tensorflow [5] and is composed of an input layer, four 3D convolutional layers each followed by batch normalization, a flattening layer, and an output layer of two class scores (Fig S1.4). Dropout is applied to the entire network at a rate of 50%. The architecture of the photorealistic rendering network is identical to the 3DCNN except that its output layer contains three neurons to which no softmax filter is applied.

The dimensions of the input layer are the same as those of the subvolume extracted from the micro-CT data and are selected according to the resolution of the training volume. The network applied to the carbon phantom scroll has an input layer of size $34 \times 34 \times 34$ voxels, which corresponds to $0.40 \times 0.40 \times 0.40mm^3$. For the lunate sigma volume, we increase the size of our subvolume inputs to $48 \times 96 \times 96$, which corresponds to $0.06 \times 0.12 \times 0.12mm^3$. While a cubic subvolume would have been preferable, the additional voxels proved to be prohibitively large for our computational environment. We chose to limit the depth of the subvolume into the substrate, as we theorize that the ink signal is primarily distributed along the substrate's surface.

The first convolutional layer has 32 filters, with subsequent layers containing half as many filters as the previous layer. All filters are of size $3 \times 3 \times 3$ and are initialized using Xavier initializations. Convolutions are performed with a stride of two voxels such that a convolutional layer's output volume is half the

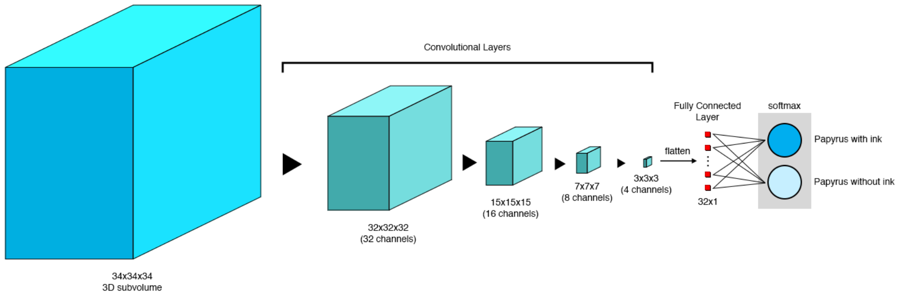

Fig S1.4. Final network layout for the ink detection network. The photorealistic rendering network is identical except for its final output layer, which contains three output neurons to which softmax is not applied.

size of its input volume along each dimension, except for the first convolution which is performed with a stride of one voxel.

After the convolutional layers, the remaining tensor values are flattened and provided as input to the fully-connected layer, which is connected to the output layer of class scores. For the 3DCNN, the softmax function is used to convert the class scores into a probability distribution indicating the presence of ink.

Grid training A significant challenge in training our model is the relative lack of training data for carbon ink detection. Machine learning methods perform best when they are provided with a large and diverse library of training samples [6]. For our purposes, a micro-CT training volume must have labels indicating the presence of carbon ink and should have been scanned at a sufficiently high resolution. We are not aware of any preexisting datasets that meet these requirements, and, for this reason, most of our experiments are trained and predicted on the carbon phantom scroll data.

When using a single dataset for both training and prediction, it is important that the data be split into non-overlapping sets. This ensures that the network captures a generalized understanding of the carbon ink signal rather than memorizing what it has already seen. However, we want to know how the network performs on the entire carbon phantom scroll, so omitting any data from our prediction set is not desirable. In order to avoid mixing our training and prediction sets while still maintaining the ability to evaluate our network for the entire scroll, we use a modified k-fold cross-validation technique which we call "grid training."

A standard k-fold cross-validation partitions the total set of shuffled subvolumes into k subsets, isolating one subset as the evaluation subset and using the others as training subsets. This method, however, is not ideal for our application. Because they are generated from adjacent surface points, the subvolumes fed into our network often contain a large amount of overlapping data. When the training data is shuffled, the overlapping subvolume information will result in the mixing of training and prediction data that we are trying to avoid. Also,

the final output from applying our method is an image of the scroll's surface, revealing the presence of ink. Shuffling the total set of subvolumes produces a sparse prediction image that is difficult to interpret visually.

Our grid training method avoids these problems by partitioning the scroll's surface spatially into k rectangular regions-of-interest. Though we call this technique grid training after the grid layout of the carbon phantom scroll, in practice it is not required that the regions actually be organized into a grid or that they span the entire 2D space. Regions of any position and size may be used. We then proceed quite similarly to a standard k-fold cross-validation procedure.

The surface points from one of these regions are reserved as a prediction and evaluation region, while the network is trained on the shuffled subvolumes from the remaining regions. Once trained, the network is applied to the reserved region to produce an ink prediction image. A subset of these points is also reserved as the evaluation set for the training run. This process is repeated k times, cycling through all regions, to produce k different networks and prediction images. We combine the prediction images from all training runs to obtain a final, full prediction image showing the network's performance across the entire dataset.

Metrics For evaluating the predictive power of the ink detection network, we used the Area Under the Receiver Operating Curve (AUC) metric and the softmax cross entropy loss function. For the photorealistic rendering network, the Huber loss function was used [7]. All metrics were taken from the performance on the evaluation set for each experiment.

Network configuration and hyperparameters

After settling on a 3DCNN approach for our methods, we performed multiple experiments to validate and optimize the network architecture and hyperparameters. These experiments were all performed on column 2 of the carbon phantom scroll and all isolated the third character (theta) as the evaluation and prediction set while training on the other 4 characters. We first performed a baseline experiment with the following parameters:

- Subvolume size: $34 \times 34 \times 34$ voxels
- Nearest neighbor subvolume interpolation
- Dropout rate: 0.5
- Learning rate: 0.001
- Minibatch size: 30
- Total training batches: 100,000 (49% of 1 complete epoch)
- Evaluation samples: 5,000
- Batch normalization after each convolutional layer
- Batch normalization momentum: 0.9
- Data augmentation (rotations, flips, etc.)

- Unnormalized subvolumes
- Adam optimizer [8]
- Xavier initializations

We then experimented with a number of these parameters, changing only one parameter at a time from the baseline experiment. The results of this experimentation are shown in Table S1.1. All results validated the baseline hyperparameters of the network.

Subvolume size and shape We performed a series of experiments to determine the ideal input subvolume size and shape for the carbon phantom scroll. Varying the shape of the subvolume changes what the network is capable of learning about the ink characteristics. If it is too small, the network may not have enough information to characterize the ink signal. If it is too large, then the network will perform slowly and may learn features unrelated to the ink. Finding a balance between size and performance is critical to perfecting the network.

Fig S1.5 shows the results of experimenting with cubic subvolumes of varying sizes. All subvolumes in this experiment were zero-padded to a side length of 44 so that the number of trainable network parameters would remain constant across trials. Larger subvolumes increased the predictive power of the classifier, as demonstrated by the AUC metric. However, larger subvolumes also require more computation and become impractical for large training libraries. We seek a balance between the two, where the network performs well and can train within a reasonable timeframe.

In addition to the general size of an input subvolume, we experimented with the shape of the subvolume along different axes. We refer to a subvolume's z dimension as its depth, since this affects how far into a substrate layer the subvolume will penetrate. We refer to a subvolume's x and y dimensions jointly as its width, since these values determine the size of the region along the substrate surface that will be visible to the network. By varying the shape of the subvolume, we capture different spatial properties of the ink's signal.

The experimental results are in line with our intuitive expectations. Additional depth proves useful to the network until it is roughly equal to the thickness of a substrate layer (Fig S1.6). Increasing the subvolume width continues to improve network performance up to a greater limit (Fig S1.7).

Our final network uses a cubic subvolume with a side length of 34 voxels. This shape was chosen because it seemed to form an optimal intersection between accuracy and runtime cost.

Simulating resolution Acquiring micro-CT scans for this work, particularly of authentic Herculaneum material, can be quite challenging. This difficulty grows as the desired scan resolution increases due to the availability, cost, and scan volume size of high resolution micro-CT scanners. Therefore, we conducted experiments to determine the viability of our method across a variety of effective resolutions.

Table S1.1. Hyperparameter tuning results.

**Experiment description**	**AUC**	**Δ(%)**	**Loss**	**Δ(%)**
Baseline	0.80	–	0.58	–
Snap to axis aligned	0.81	1.25	0.62	6.90
Interpolated	0.82	2.50	0.53	-8.62
Dropout rate 0.0	0.81	1.25	0.61	5.17
Dropout rate 0.1	0.83	3.75	0.55	-5.17
Dropout rate 0.2	0.82	2.50	0.62	6.90
Dropout rate 0.3	0.77	-3.75	0.69	18.97
Dropout rate 0.4	0.82	2.50	0.61	5.17
Dropout rate 0.6	0.80	0.00	0.57	-1.72
Dropout rate 0.7	0.77	-3.75	0.59	1.72
Dropout rate 0.8	0.80	0.00	0.56	-3.45
Dropout rate 0.9	0.76	-5.00	0.63	8.62
Learning rate 0.1	0.50	-37.50	0.74	27.59
Learning rate 0.01	0.77	-3.75	0.72	24.14
Learning rate 0.0001	0.79	-1.25	0.63	8.62
Learning rate 0.00001	0.69	-13.75	0.64	10.34
Learning rate 0.000001	0.52	-35.00	0.71	22.41
No batch normalization	0.50	-37.50	0.69	18.97
Batch normalization momentum 0.999	0.79	-1.25	1.11	91.38
Batch normalization momentum 0.99	0.80	0.00	0.59	1.72
Batch normalization momentum 0.8	0.80	0.00	0.59	1.72
Batch normalization momentum 0.7	0.80	0.00	0.57	-1.72
Batch normalization momentum 0.6	0.79	-1.25	0.60	3.45
Batch normalization momentum 0.5	0.78	-2.50	0.62	6.90
Minibatch size 4	0.77	-3.75	0.61	5.17
Minibatch size 8	0.79	-1.25	0.62	6.90
Minibatch size 16	0.80	0.00	0.61	5.17
Minibatch size 32	0.80	0.00	0.57	-1.72
Minibatch size 64	0.81	1.25	0.55	-5.17
3 epochs	0.83	3.75	0.56	-3.45
No augmentation	0.81	1.25	0.54	-6.90
Normalized subvolumes	0.82	2.50	0.55	-5.17
Adagrad optimizer	0.65	-18.75	0.66	13.79

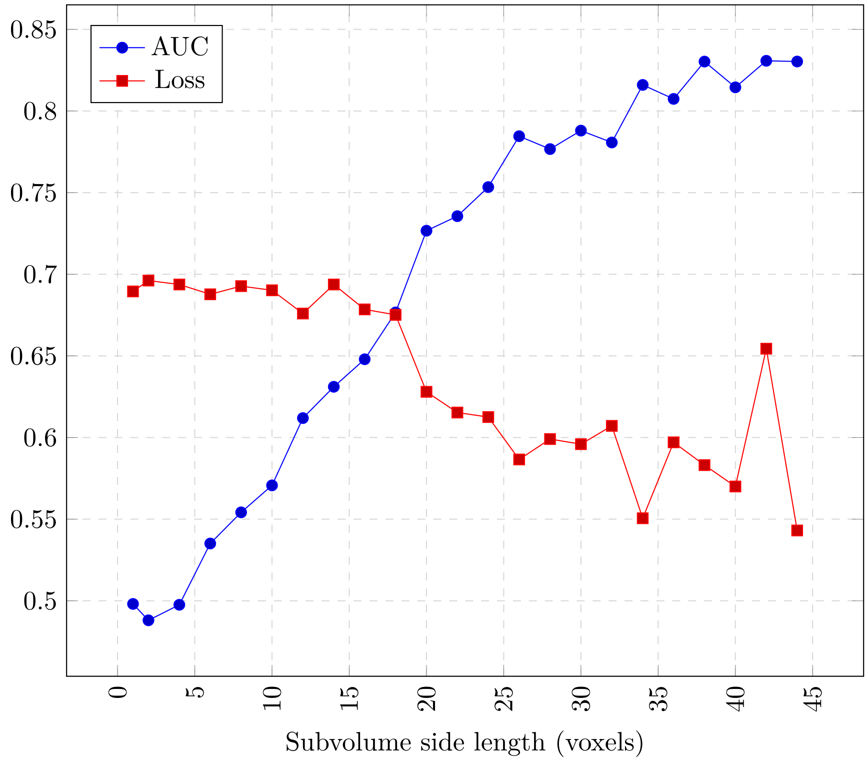

Fig S1.5. Network performance as a function of cubic subvolume side length.

We used bicubic interpolation to downsample the carbon phantom volume to various simulated resolutions and then upsample them to the original dimensions. We then apply the machine learning pipeline to each of these volumes individually and compare their performance metrics. This experiment helps us understand the signal as a function of resolution, which is a step beyond knowing that lower resolution will dampen the signal.

Fig S1.8 shows that the predictive power of the classifier declines roughly linearly as the simulated resolution decreases.

Designing a custom micro-CT scanner

The requirements for our ideal micro-CT scanner are the ability to evaluate a Herculaneum scroll at a 5 micron voxel size. Using previously studied Herculaneum scrolls as a target, we can bound the dimensions of such a scroll at 150mm long with a 100mm diameter.

The length of the sample should not pose a problem: with sufficient transaxial travel of the sample stage, the entire scroll can be scanned. One approach

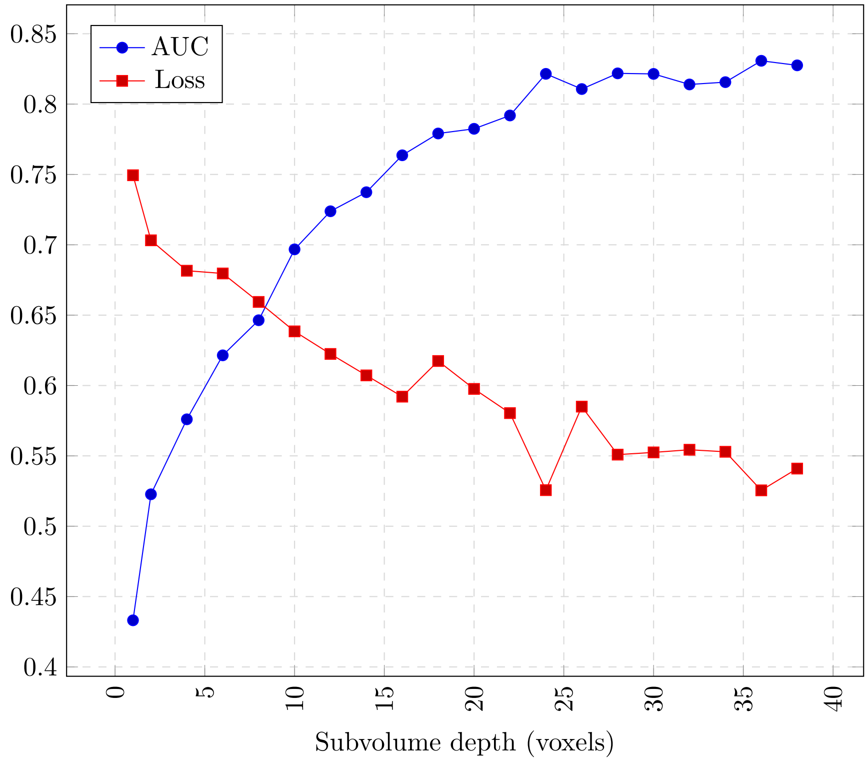

Fig S1.6. Network performance as a function of subvolume depth. Width was held constant at 48 voxels.

is to perform multiple, offset micro-CT scans using a circular trajectory around the scroll. These sub-scans can be stitched together after reconstruction in a straightforward manner [9, 10]. Alternatively, a helical trajectory can achieve the same reconstruction volume in one long scan. When using an exact reconstruction algorithm, the helical trajectory holds the advantage of eliminating cone-beam artifacts. The mechanical accuracy required of the system is more demanding with this setup, but can be achieved nonetheless.

As previously stated, the key challenge in reaching a high enough resolution for a 100mm diameter sample is capturing enough pixels horizontally in the projection images. With ideal positioning, achieving a 5 micron voxel size requires a minimum of 20,000 pixels across the sample. Using triple off-setting (tiling) of a 16 megapixel CCD sensor, the Bruker SkyScan 1272 would be able to capture 14,450 pixels across the sample. This setup would generate a volume with a 6.92 voxel size. In principle, this approach can be extended by tiling the detector four times to reach a 5 micron voxel size.

The stability of the x-ray emission point is key to achieving effective results. Because the higher spatial resolution will result in extended acquisition times,

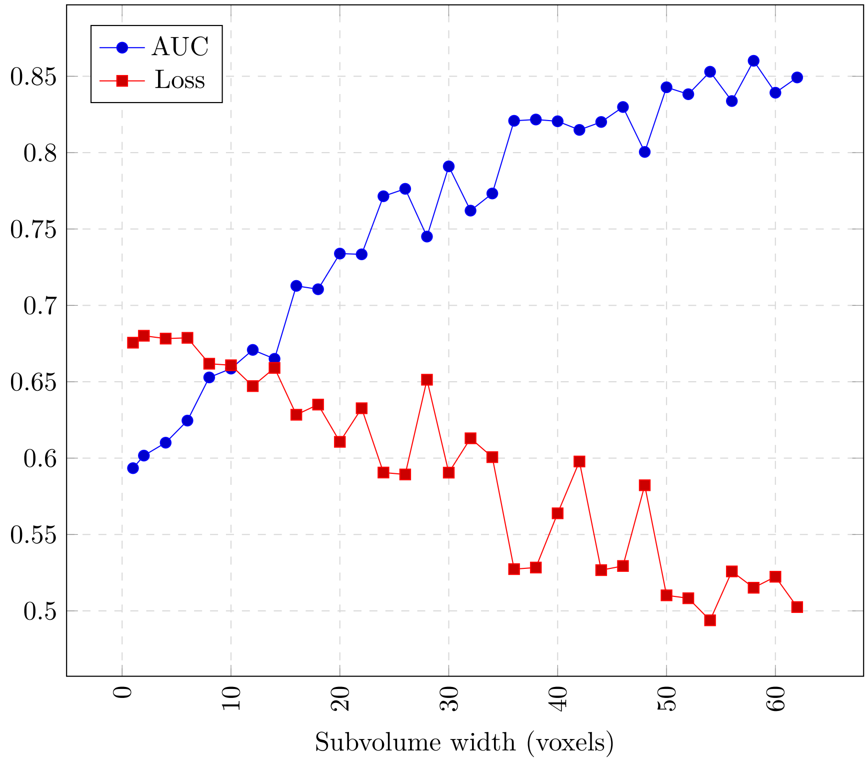

Fig S1.7. Network performance as a function of subvolume width. Depth was held constant at 24 voxels.

this puts further constraints on the x-ray source hardware requirements. In order to reach the required spatial resolution, the x-ray spot size of a cone or fan beam tube needs to be below our lower resolution limit of 5 micron. A large number of hardware options can achieve this spot size, including both open and closed, reflection and transmission laboratory x-ray sources.

Alternative methods

In our previous work on the En-Gedi scroll, we extracted a 1D vector of voxels, normal to a surface point, and applied a maximum intensity filter to render the ink visible [11]. Before arriving at a method based on a 3DCNN, we first tried more immediate extensions to this existing method. We present here the results of applying these methods to the carbon phantom scroll. While none of these alternative methods showed promise as standalone ink detectors, they did provide valuable insight into the complexities of carbon ink signals. In particular, the structural patterns of the papyrus substrate dominated the outputs of these filters. It became clear that a more general carbon ink detector

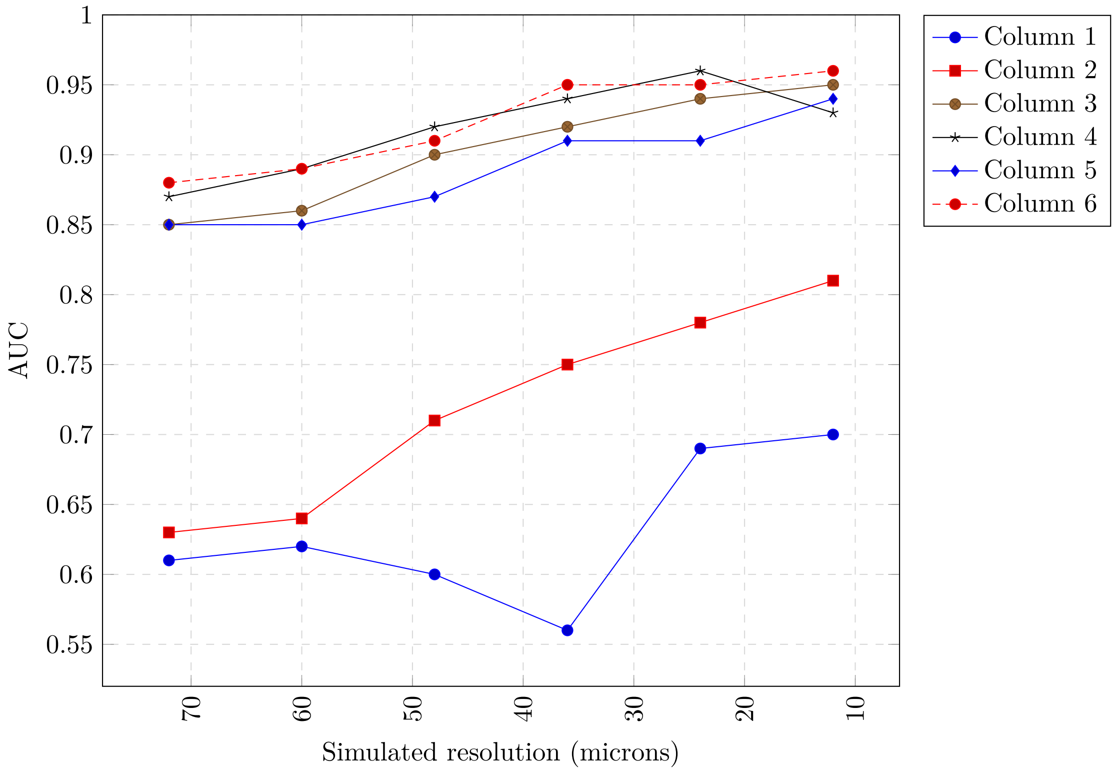

Fig S1.8. Network performance as a function of simulated CT resolution.

would require an analytical model that could better account for the extreme local variance of the papyrus structure.

Machine learning with voxel vectors Rather than applying a simple filter to a voxel vector, as in previous work, we trained a small 1DCNN to detect ink signal using the voxel vector. A 1D voxel vector of length 17 is first extracted along the surface normal vector at the point, extending both in the positive and negative directions. The input vector is fed to a neural network with two 1D convolutional layers, each followed by batch normalization. The convolution kernel size is 3 and the stride is 2. The output from these convolutional layers is fed to a fully connected layer, dropout is applied, and the resulting two binary classification values are processed identically to the way they are handled in our final methods. Fig S1.9 shows the results of this method applied to the carbon phantom scroll.

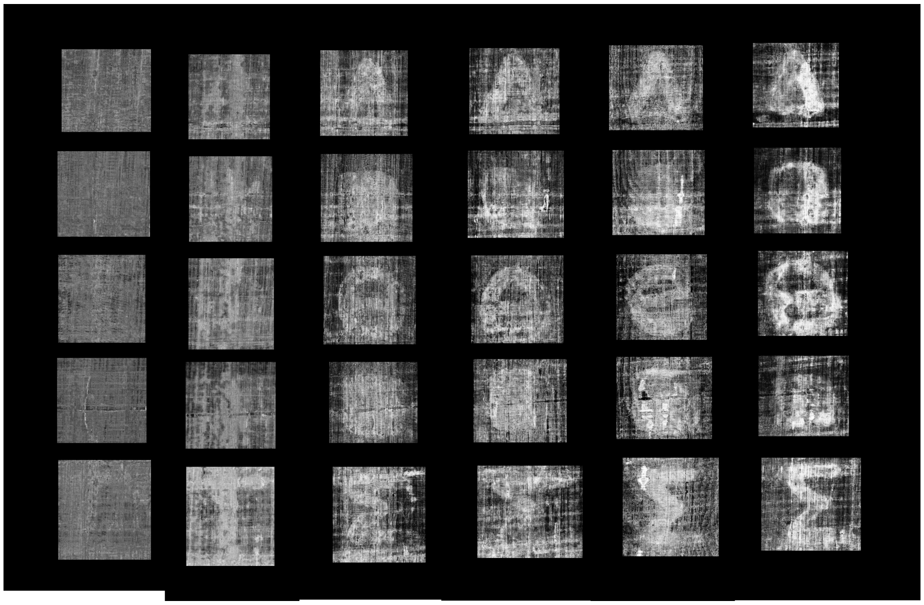

Fig S1.9. The 1DCNN voxel vector method applied to the carbon phantom scroll. Each column was an independent 5-fold cross-validation experiment. The method shows promise at revealing carbon ink as far as column 3 in the scroll but does not perform as well in columns 1 or 2.

Descriptive statistics of subvolumes The maximum intensity filter applied during the En-Gedi work is one of a number of previously developed filters based upon the descriptive statistics (Minimum, Maximum, Mean, Standard Deviation, Median, and Variance). We ran an experiment to determine if one of these filters would reveal the ink if they were provided full subvolumes rather than a voxel vector. If this experiment performed well, it would indicate that the carbon ink signal is not subtle or complex, but rather that it forms a dominant statistical pattern over a wide region-of-support. The resulting values for each subvolume were not fed into a neural network, but were simply plotted on a texture image. Fig S1.10 shows the results of the median filter, which was the most successful of those tried. While the ink is made more visible in some of the columns, the output is dominated by pattern of the papyrus fibers.

Machine learning with descriptive statistics While the previous method explored the descriptive statistics in isolation, we also explored whether the statistics for a subvolume could be combined by a neural network to predict the presence of ink. For each subvolume, we first calculate the six previously described descriptive statistics. We then provide these values as the input vector to a simple neural network. The network has two fully connected layers of 20 neurons each and an output layer which produces a pixel-wise classification of "ink" or "not ink." Fig S1.11 shows the results of this method. While this

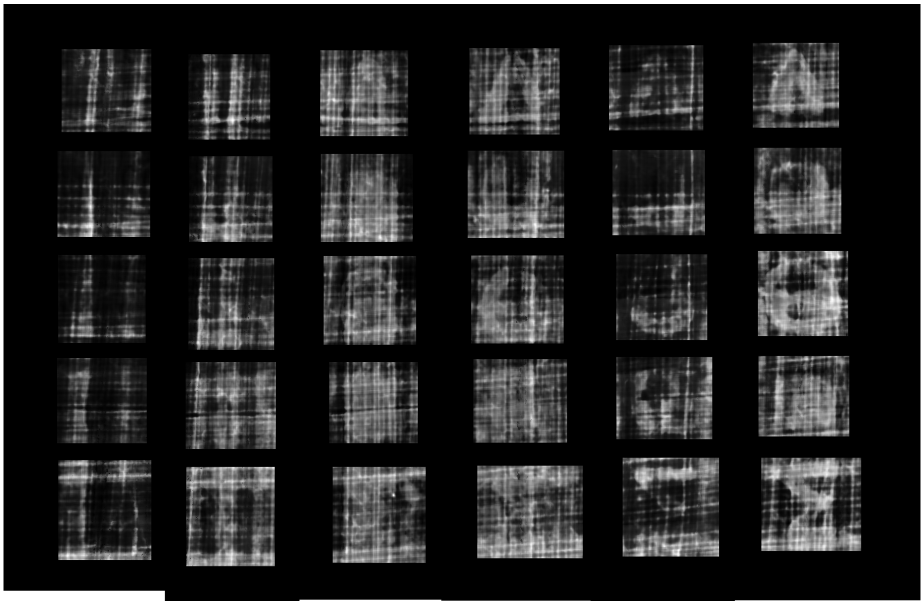

Fig S1.10. Median filter applied to subvolumes of the carbon phantom scroll. The filter is able to reveal a faint signal of ink in column 3 but not columns 1 or 2.

network does show some ability to detect and clarify carbon ink, the results are inconsistent.

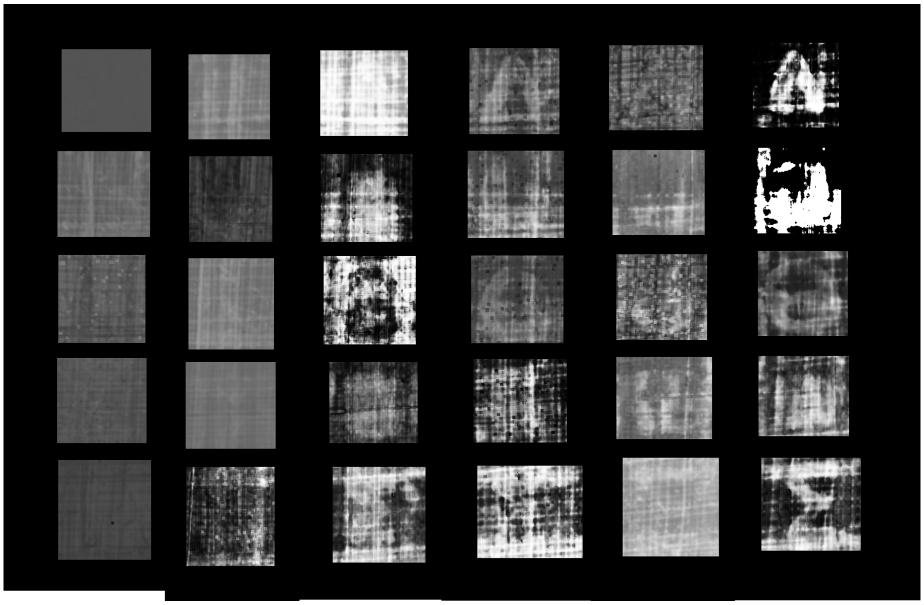

Fig S1.11. Descriptive Statistics Neural Network filter on the carbon phantom scroll. Each column was an independent 5-fold cross-validation experiment. As with other early experiments, there is not a detectable ink signal below column 3.

References

[1] Valette S, Chassery JM. Approximated centroidal voronoi diagrams for uniform polygonal mesh coarsening. In: Computer Graphics Forum. vol. 23. Wiley Online Library; 2004. p. 381–389.

[2] Sheffer A, Lévy B, Mogilnitsky M, Bogomyakov A. ABF++: fast and robust angle based flattening. ACM Transactions on Graphics (TOG). 2005;24(2):311–330.

[3] Krizhevsky A, Sutskever I, Hinton GE. Imagenet classification with deep convolutional neural networks. In: Advances in neural information processing systems; 2012. p. 1097–1105.

[4] Yoo TS, Ackerman MJ, Lorensen WE, Schroeder W, Chalana V, Aylward S, et al. Engineering and algorithm design for an image processing API: a technical report on ITK-the insight toolkit. Studies in health technology and informatics. 2002; p. 586–592.

[5] Abadi M, Barham P, Chen J, Chen Z, Davis A, Dean J, et al. TensorFlow: A System for Large-Scale Machine Learning. In: OSDI. vol. 16; 2016. p. 265–283.

[6] Halevy A, Norvig P, Pereira F. The unreasonable effectiveness of data. IEEE Intelligent Systems. 2009;24(2):8–12.

[7] Huber PJ, et al. Robust estimation of a location parameter. The annals of mathematical statistics. 1964;35(1):73–101.

[8] Kingma DP, Ba J. Adam: A method for stochastic optimization. arXiv preprint arXiv:14126980. 2014;.

[9] Kyrieleis A, Ibison M, Titarenko V, Withers P. Image stitching strategies for tomographic imaging of large objects at high resolution at synchrotron sources. Nuclear Instruments and Methods in Physics Research Section A: Accelerators, Spectrometers, Detectors and Associated Equipment. 2009;607(3):677–684.

[10] Ji C. Accurate 3D data stitching in circular cone-beam micro-CT. Journal of X-ray science and technology. 2010;18(2):99–110.

[11] Seales WB, Parker CS, Segal M, Tov E, Shor P, Porath Y. From damage to discovery via virtual unwrapping: Reading the scroll from En-Gedi. Science Advances. 2016;2(9). doi:10.1126/sciadv.1601247.
